# Variable-Length Resolvability for General Sources and Channels†

**DOI:** 10.3390/e25101466

**Published:** 2023-10-19

**Authors:** Hideki Yagi, Te Sun Han

**Affiliations:** 1 Department of Computer and Network Engineering, The University of Electro-Communications, Tokyo 182-8585, Japan; 2 The National Institute of Information and Communications Technology (NICT), Tokyo 184-8795, Japan

**Keywords:** random number generation, source resolvability, channel resolvability, output approximation, variable-length resolvability

## Abstract

We introduce the problem of variable-length (VL) source resolvability, in which a given target probability distribution is approximated by encoding a VL uniform random number, and the asymptotically minimum average length rate of the uniform random number, called the VL resolvability, is investigated. We first analyze the VL resolvability with the variational distance as an approximation measure. Next, we investigate the case under the divergence as an approximation measure. When the asymptotically exact approximation is required, it is shown that the resolvability under two kinds of approximation measures coincides. We then extend the analysis to the case of channel resolvability, where the target distribution is the output distribution via a general channel due to a fixed general source as an input. The obtained characterization of channel resolvability is fully general in the sense that, when the channel is just an identity mapping, it reduces to general formulas for source resolvability. We also analyze the second-order VL resolvability.

## 1. Introduction

Generating a random number subject to a given probability distribution has a number of applications, such as in information security, statistical machine learning, and computer science. From the viewpoint of information theory, random number generation may be considered to be a transformation (encoding) of sequences emitted from a given source with *coin distribution* into other sequences with *target distribution* via a deterministic mapping [1,2,3]. Among others, there have been two major types of problems of random number generation: *intrinsic randomness* [4,5] and (source) *resolvability* [6,7]. In the former case, a fixed-length (FL) uniform random number is extracted from an arbitrary coin distribution, and we want to find the *maximum* achievable rate of such uniform random numbers. In the latter case, in contrast, an FL uniform random number used as a coin distribution is encoded to approximate a given *target distribution*, and we want to find the *minimum* achievable rate of such uniform random numbers. Thus, there is a duality between these two problems.

The problem of intrinsic randomness has been extended to the case of *variable-length* (VL) uniform random numbers, for which the length of random numbers may vary. This problem, referred to as the *VL intrinsic randomness*, was first introduced by Vembu and Verdú [5] for a finite source alphabet and later extended by Han [4] to a countably infinite alphabet. This problem was actually motivated because, in many practical situations, it is indispensable to consider cases where FL uniform random numbers are not available, and, instead, *VL uniform random numbers* are available; typically, this is in cases where we work with Elias’ universal random numbers [1]. The use of such uniform random numbers is expected generally to increase the achievable average length rate for intrinsic randomness. Then, the following natural question may be raised: Can we indeed lower the average length rate needed in the “resolvability” problem by using VL random numbers? The answer is “yes”. Despite the duality between these two kinds of problems for random number generation, the VL counterpart in the resolvability problem has not been discussed, where we focus on this problem.

We introduce the problem of *VL source/channel resolvability*, where a given target probability distribution is to be approximated by encoding a *VL uniform random number*. Distance measures between the target distribution and an approximated distribution are used to measure the fineness of the approximation. We first analyze the fundamental limit on VL source resolvabilities with the variational distance as an approximation measure in Section 3. We use the smooth Shannon entropy, which is a version of smooth Rényi entropy [8], to characterize the δ-source resolvability, which is defined as the minimum achievable length rate of uniform random numbers with an asymptotic distance of less than or equal to δ∈[0,1). In the proof of the direct part, we will develop a simple version of *information spectrum slicing* [2], in which each “sliced” information density quantized to an integer is approximated by an FL uniform random number. Due to the simplicity of the method, the analysis with variational distance is first facilitated. As an important implication of general formulas for the δ-source resolvability, it is shown that the minimum resolvability rate of VL resolvability is equal to (1−δ) times that of FL resolvability when the source is stationary and memoryless or is even with one-point spectrum (cf. Corollary 1). This result indicates an advantage of the use of a VL uniform random number when δ>0 because we can make the VL resolvability rate strictly smaller than an FV one. We then extend these analyses to the case under the (unnormalized) divergence as an approximation measure in Section 4. When δ=0, that is, when the asymptotically exact approximation is required, it is shown that the 0-source resolvabilities under two kinds of approximation measures coincide with each other.

In Section 5, we then consider the problem of *channel resolvability* [6,9,10], in which not only a source but also a channel is fixed, and the output distribution via the channel is now the target of approximation. This problem, also referred to as the problem of output approximation, provides a powerful tool to analyze the fundamental limits of various problems in information theory and information security. Some such examples include identification codes [11,12,13], distributed hypothesis testing [14], message authentication [15], secret key generation [16], and coding for secure communication [17,18,19]. We consider two types of problems in which either a general source (*mean-channel resolvability*) or a VL uniform random number (*VL channel resolvability*) is used as a coin distribution. It is shown that the formulas established are equal for both coin distributions. In the special case that the channel is the identity mapping, the formulas established reduce to those in source resolvability as established in Section 3 and Section 4.

From Section 3, Section 4 and Section 5, the so-called *first-order* resolvability rates are analyzed, and the next important step may be the second-order analysis. Second-order analyses for various coding problems were initiated by Strassen [20] and have been studied in the past decade or so (cf. [21,22,23,24,25,26,27,28,29,30,31]). We also analyze the second-order fundamental limits of the VL channel/source resolvability in Section 6. In this paper, it is shown that the VL δ-*source* resolvability under the variational distance is equal to the minimum achievable rate of *fixed-to-variable-length source codes* with an error probability of less than or equal to δ. It is demonstrated that this close relationship provides a single-letter characterization for the first- and second-order source resolvability under the variational distance when the source is stationary and memoryless. It is worth noting that second-order analyses for the VL setting are relatively few, compared to those in the FL setting. The second-order formulas established in this paper are of importance from this perspective, too.

The remainder of the paper is organized as follows: Section 2 reviews the problem of FL source resolvability and the relations between the minimum resolvability rate and the minimum coding rate of FL source codes. Section 3 formally introduces the problem of VL source resolvability with variational distance as an approximation measure. Then, Section 4 discusses VL source resolvability with divergence as an approximation measure, and Section 5 generalizes the settings to channel resolvability. Section 6 investigates the second-order fundamental limits of the VL channel/source resolvability. Section 7 concludes the paper with a discussion of possible extensions.

## 2. FL Resolvability: Review

Let U={1,2,…,K} be a finite alphabet of size *K*, and let X be a finite or countably infinite alphabet. Let $X={\left\{X^{n}\right\}}_{n=1}^{\infty }$ be a *general source* [2], where $P_{X^{n}}$ is a probability distribution on $X^{n}$. We do not impose any assumptions, such as stationarity or ergodicity. In this paper, we identify $X^{n}$ with its probability distribution $P_{X^{n}}$, and these symbols are used interchangeably.

**Figure 1:**
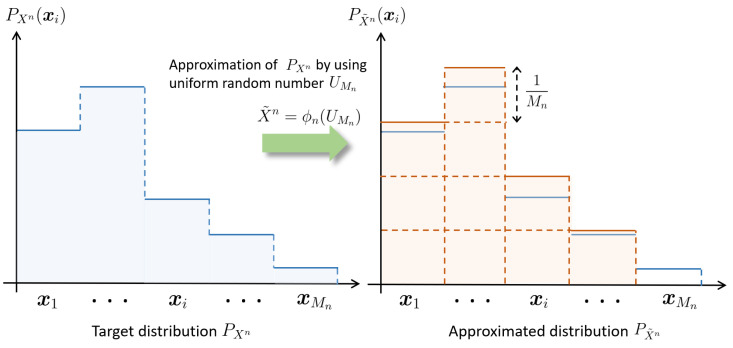
Illustration of the problem of FL resolvability.

We first review the problem of *FL* (source) resolvability [2] using the variational distance as an approximation measure. Let $U_{M_{n}}$ denote the *uniform random number*, which is a random variable *uniformly* distributed over $U_{M_{n}}:=\left\{1,\ldots ,M_{n}\right\}$. Consider the problem of approximating the *target distribution* $P_{X^{n}}$ by using $U_{M_{n}}$ as a *coin distribution* via a deterministic mapping $\varphi_{n}:\left\{1,\ldots ,M_{n}\right\}\to X^{n}$. Denoting ${\tilde{X}}^{n}=\varphi_{n}\left(U_{M_{n}}\right)$, we want to make $P_{{\tilde{X}}^{n}}$ approximate $P_{X^{n}}$ (cf. Figure 1). A standard choice of the performance measure for approximation is

(1) $$\begin{array}{c} d\left(P_{X^{n}},P_{{\tilde{X}}^{n}}\right):=\frac{1}{2}\sum_{x\in X^{n}}\left|P_{X^{n}}\left(x\right)-P_{{\tilde{X}}^{n}}\left(x\right)\right|, \end{array}$$

which is referred to as the *variational distance* between $P_{X^{n}}$ and $P_{{\tilde{X}}^{n}}$. It is easily seen that

(2) $$\begin{array}{cc} 0 & \leq d(P_{X^{n}},P_{{\tilde{X}}^{n}})\leq 1. \end{array}$$

Let us now review the problem for *source resolvability*. Throughout this paper, logarithms are of the base *K*.

**Definition** **1** (FL resolvability)**.** *A resolution rate R≥0 is said to be FL achievable or simply f-achievable (under the variational distance) if there exists a deterministic mapping $\varphi_{n}:\left\{1,\ldots ,M_{n}\right\}\to X^{n}$ satisfying*

(3) $$\begin{array}{cc} \underset{n\to \infty }{lim sup}\frac{1}{n}\log M_{n} & \leq R, \end{array}$$

(4) $$\begin{array}{cc} \lim_{n\to \infty }d\left(P_{X^{n}},P_{{\tilde{X}}^{n}}\right) & =0, \end{array}$$

*where ${\tilde{X}}^{n}=\varphi_{n}\left(U_{M_{n}}\right)$ and $U_{M_{n}}$ is the uniform random number over $U_{M_{n}}$. The infimum of f-achievable rates, i.e.,*

(5) $$\begin{array}{c} S_{f}\left(X\right):=\inf\left\{R:\;R\;is\;f-achievable\right\} \end{array}$$

*is called the FL resolvability or simply f-resolvability.*

Then, we have the following theorem:

**Theorem** **1** (Han and Verdú [6])**.** *For any general target source X,*

(6) $$\begin{array}{c} S_{f}\left(X\right)=\bar{H}\left(X\right), \end{array}$$

*where*

(7) $$\begin{array}{cc} \bar{H}\left(X\right) & :=\inf\left\{a:\lim_{n\to \infty }\mathrm{Pr}\left\{\frac{1}{n}\log\frac{1}{P_{X^{n}}\left(X^{n}\right)}>a\right\}=0\right\}. \end{array}$$

**Remark** **1.** *As a dual counterpart of (7), we may define*

(8) $$\begin{array}{cc} \underline{H}\left(X\right) & :=\sup\left\{b:\lim_{n\to \infty }\mathrm{Pr}\left\{\frac{1}{n}\log\frac{1}{P_{X^{n}}\left(X^{n}\right)}<b\right\}=0\right\}. \end{array}$$

*Sources such that $\bar{H}\left(X\right)=\underline{H}\left(X\right)$ are called one-point spectrum sources (or equivalently, said to satisfy the strong converse property (cf. Han [2])), which includes stationary memoryless sources and stationary ergodic sources, etc. This class of sources is discussed later in Corollary 1.*

The following problem is called the *δ-resolvability problem* [2,7], which relaxes the condition on the variational distance, compared to (4).

**Definition** **2** (FL δ-resolvability)**.** *For a fixed δ∈[0,1), a resolution rate R≥0 is said to be FL δ-achievable or simply f(δ)-achievable (under the variational distance) if there exists a deterministic mapping $\varphi_{n}:\left\{1,\ldots ,M_{n}\right\}\to X^{n}$ satisfying*

(9) $$\begin{array}{cc} \underset{n\to \infty }{lim sup}\frac{1}{n}\log M_{n} & \leq R, \end{array}$$

(10) $$\begin{array}{cc} \underset{n\to \infty }{lim sup}d\left(P_{X^{n}},P_{{\tilde{X}}^{n}}\right) & \leq \delta , \end{array}$$

*where ${\tilde{X}}^{n}=\varphi_{n}\left(U_{M_{n}}\right)$ and $U_{M_{n}}$ is the uniform random number over $U_{M_{n}}$. The infimum of all f(δ)-achievable rates, i.e.,*

(11) $$\begin{array}{c} S_{f}\left(\delta |X\right):=\inf\left\{R:\;R\;is\;f(\delta )-achievable\right\} \end{array}$$

*is referred to as the FL δ-resolvability or simply f(δ)-resolvability.*

Then, a characterization of $S_{f}\left(\delta |X\right)$ is given by

**Theorem** **2** (Steinberg and Verdú [7])**.** *For any general target source X,*

(12) $$\begin{array}{c} S_{f}\left(\delta |X\right)={\bar{H}}_{\delta }\left(X\right)\;\;\;\;\left(\delta \in \left[0,1\right)\right), \end{array}$$

*where*

(13) $$\begin{array}{cc} {\bar{H}}_{\delta }\left(X\right) & :=\inf\left\{a:\underset{n\to \infty }{lim sup}\mathrm{Pr}\left\{\frac{1}{n}\log\frac{1}{P_{X^{n}}\left(X^{n}\right)}>a\right\}\leq \delta \right\}. \end{array}$$

**Remark** **2.** *The FL resolvability problem is deeply related to the FL source coding problem allowing a probability of a decoding error up to ε. Denoting by $R_{f}\left(\varepsilon |X\right)$ the minimum achievable rate for the source X, there is the relationship [7]:*

(14) $$\begin{array}{c} R_{f}\left(\varepsilon |X\right)={\bar{H}}_{\varepsilon }\left(X\right)\;\;\;\left(\forall \varepsilon \in \left[0,1\right)\right) \end{array}$$

*and, hence, by Theorem 2,*

(15) $$\begin{array}{c} S_{f}\left(\delta |X\right)=R_{f}\left(\delta |X\right)\;\;\;\left(\forall \delta \in \left[0,1\right)\right). \end{array}$$

*Formula (14) can also be shown with a smooth Rényi entropy of order zero [32].*

## 3. VL Resolvability: Variational Distance

In this section, we introduce the problem of *variable-length* (VL) resolvability, where the target probability distribution is approximated by encoding a *VL uniform random number*. As an initial step, we analyze the fundamental limit on the VL resolvability with the variational distance as an approximation measure.

### 3.1. Definitions

Let $U^{*}$ denote the set of all sequences $u\in U^{m}$ over m=0,1,2,⋯, where $U^{0}=\left\{\lambda \right\}$ (λ is the null string). Let $L_{n}$ denote a random variable which takes a value in {0,1,2,…}. We define the *VL uniform random number* $U^{\left(L_{n}\right)}$ so that $U^{\left(m\right)}$ is uniformly distributed over $U^{m}$ given $L_{n}=m$. In other words,

(16) $$\begin{array}{cc} P_{U^{\left(L_{n}\right)}}\left(u,m\right) & :=\mathrm{Pr}\left\{U^{\left(L_{n}\right)}=u,L_{n}=m\right\}=\frac{\mathrm{Pr}\{L_{n}=m\}}{K^{m}}\;\;\;\;\left(\forall u\in U^{m}\right), \end{array}$$

(17) $$\begin{array}{cc} \mathrm{Pr}\{U^{\left(L_{n}\right)}=u|L_{n}=m\} & =\frac{P_{U^{\left(L_{n}\right)}}\left(u,m\right)}{\mathrm{Pr}\{L_{n}=m\}}=\frac{1}{K^{m}}\;\;\;\;\left(\forall u\in U^{m}\right), \end{array}$$

where K=|U|. It should be noticed that the VL sequence $u\in U^{m}$ is generated with the joint probability $P_{U^{\left(L_{n}\right)}}\left(u,m\right)$.

We formally define the δ-resolvability problem under the variational distance using the VL random number, called the *VL δ-resolvability* or simply v(δ)-*resolvability*.

**Definition** **3** (VL δ-resolvability: variational distance)**.** A resolution rate R≥0 is said to be VL δ-achievable (under the variational distance) with δ∈[0,1) if there exists a VL uniform random number $U^{\left(L_{n}\right)}$ and a deterministic mapping $\varphi_{n}:U^{*}\to X^{n}$ satisfying

(18) $$\begin{array}{cc} \underset{n\to \infty }{lim sup}\frac{1}{n}E\left[L_{n}\right] & \leq R, \end{array}$$

(19) $$\begin{array}{cc} \underset{n\to \infty }{lim sup}d\left(P_{X^{n}},P_{{\tilde{X}}^{n}}\right) & \leq \delta , \end{array}$$

*where E[·] denotes the expected value and ${\tilde{X}}^{n}=\varphi_{n}\left(U^{\left(L_{n}\right)}\right)$. The infimum of all v(δ)-achievable rates, i.e.,*

(20) $$\begin{array}{c} S_{v}\left(\delta |X\right):=\inf\left\{R:\;R\;is\;v(\delta )-achievable\right\} \end{array}$$

*is referred to as the VL δ-resolvability or simply v(δ)-resolvability.*

*If δ=0, v(0)-achievable is said to be VL achievable or simply v-achievable (under the variational distance). The infimum of all v-achievable rates, i.e.,*

(21) $$\begin{array}{c} S_{v}\left(X\right):=\inf\left\{R:\;R\;is\;v-achievable\right\} \end{array}$$

*is called the VL resolvability or simply v-resolvability.*

**Remark** **3.** *One may think that condition (18) can be replaced with the condition on the sup-entropy rate:*

(22) $$\begin{array}{cc} \underset{n\to \infty }{lim sup}\frac{1}{n}H\left(U^{\left(L_{n}\right)}\right) & \leq R \end{array}$$

*as in [6], where H(·) denotes the Shannon entropy. Indeed, both conditions yield the same resolvability result. To see this, let us denote by ${\tilde{S}}_{v}\left(\delta |X\right)$ the infimum of v-achievable rates R under constraints (19) and (22). It is easily checked that*

(23) $$\begin{array}{cc} E[L_{n}] & =\sum_{m=1}^{\infty }\sum_{u\in U^{m}}P_{U^{\left(L_{n}\right)}}\left(u,m\right)\log K^{m}\\ \;\; & =\sum_{m=1}^{\infty }\sum_{u\in U^{m}}P_{U^{\left(L_{n}\right)}}\left(u,m\right)\log\frac{\mathrm{Pr}\{L_{n}=m\}}{P_{U^{\left(L_{n}\right)}}\left(u,m\right)}\\ \; & =H\left(U^{\left(L_{n}\right)}\right)-H\left(L_{n}\right)\leq H\left(U^{\left(L_{n}\right)}\right). \end{array}$$

*This implies $S_{v}\left(\delta |X\right)\leq {\tilde{S}}_{v}\left(\delta |X\right)$. On the other hand, by invoking the well-known relation (cf. ([33], Corollary 3.12)), it holds that*

(24) $$\begin{array}{c} H\left(L_{n}\right)\leq \log\left(e\cdot E\left[L_{n}\right]\right). \end{array}$$

*Consider any resolution rate $R>S_{v}\left(\delta |X\right)$. Then, (18) holds for some $U^{\left(L_{n}\right)}$ and $\varphi_{n}$ and, hence, (24) leads to*

(25) $$\begin{array}{c} \lim_{n\to \infty }\frac{1}{n}H\left(L_{n}\right)=\lim_{n\to \infty }\frac{1}{n}\log\left(e\cdot E\left[L_{n}\right]\right)=0. \end{array}$$

*From this equation, (23) yields that*

(26) $$\begin{array}{c} \underset{n\to \infty }{lim sup}\frac{1}{n}H\left(U^{\left(L_{n}\right)}\right)=\underset{n\to \infty }{lim sup}\frac{1}{n}E\left[L_{n}\right]\leq R \end{array}$$

*to obtain $R\geq {\tilde{S}}_{v}\left(\delta |X\right)$, implying that $S_{v}\left(\delta |X\right)\geq {\tilde{S}}_{v}\left(\delta |X\right)$. Thus, $S_{v}\left(\delta |X\right)={\tilde{S}}_{v}\left(\delta |X\right)$.*

### 3.2. Smooth Shannon Entropy

To establish a general formula for $S_{v}\left(\delta |X\right)$, we introduce the following quantity for a general source X. Let $P(X^{n})$ denote the set of all probability distributions on $X^{n}$. For δ∈[0,1), by defining the *δ-ball* using the variational distance

(27) $$\begin{array}{c} B_{\delta }\left(X^{n}\right)=\left\{P_{V^{n}}\in P\left(X^{n}\right):d\left(P_{X^{n}},P_{V^{n}}\right)\leq \delta \right\}, \end{array}$$

we introduce the *smooth Shannon entropy*:

(28) $$\begin{array}{cc} H_{\left[\delta \right]}\left(X^{n}\right) & :=\underset{P_{V^{n}}\in B_{\delta }\left(X^{n}\right)}{\inf}\sum_{x\in X^{n}}P_{V^{n}}\left(x\right)\log\frac{1}{P_{V^{n}}\left(x\right)}\\ \; & =\underset{P_{V^{n}}\in B_{\delta }\left(X^{n}\right)}{\inf}H\left(V^{n}\right), \end{array}$$

where $H(V^{n})$ denotes the Shannon entropy of $P_{V^{n}}$. The $H_{\left[\delta \right]}\left(X^{n}\right)$ is a nonincreasing function of δ. Based on this quantity for a general source $X={\left\{X^{n}\right\}}_{n=1}^{\infty }$, we define

(29) $$\begin{array}{cc} H_{\left[\delta \right]}\left(X\right) & =\underset{n\to \infty }{lim sup}\frac{1}{n}H_{\left[\delta \right]}\left(X^{n}\right). \end{array}$$

**Remark** **4.** *Renner and Wolf [8] have defined the smooth Rény entropy of order α∈(0,1)∪(1,∞) as*

(30) $$\begin{array}{c} H_{\left[\delta \right]}^{\alpha }\left(X^{n}\right)=\underset{P_{V^{n}}\in B_{\delta }\left(X^{n}\right)}{\inf}\frac{1}{1-\alpha }\log\sum_{x\in X^{n}}P_{V^{n}}{\left(x\right)}^{\alpha }. \end{array}$$

*By letting α↑1, we have*

(31) $$\begin{array}{c} \lim_{\alpha ↑1}H_{\left[\delta \right]}^{\alpha }\left(X^{n}\right)=H_{\left[\delta \right]}\left(X^{n}\right). \end{array}$$

*As for the proof, see Appendix A.*

### 3.3. General Formula for General δ∈[0,1)

The following main theorem indicates that the v(δ)-resolvability $S_{v}\left(\delta |X\right)$ can be characterized by the smooth Shannon entropy for X.

**Theorem** **3.** *For any general target source X,*

(32) $$\begin{array}{c} S_{v}\left(\delta |X\right)=\lim_{\gamma ↓0}H_{\left[\delta +\gamma \right]}\left(X\right)\;\;\;\left(\delta \in \left[0,1\right)\right). \end{array}$$

**Remark** **5.** *In Formula (32), the limit $\lim_{\gamma ↓0}$ of the offset term +γ appears in the characterization of $S_{v}\left(\delta |X\right)$. This is because the smooth entropy $H_{\left[\delta \right]}\left(X^{n}\right)$ for $X^{n}$ involves the infimum over the non-asymptotic δ-ball $B_{\delta }\left(X^{n}\right)$ for a given length n. Alternatively, we may consider the asymptotic δ-ball defined as*

(33) $$\begin{array}{c} B_{\delta }\left(X\right)=\left\{V={\left\{V^{n}\right\}}_{n=1}^{\infty }:\underset{n\to \infty }{lim sup}d\left(P_{X^{n}},P_{V^{n}}\right)\leq \delta \right\}, \end{array}$$

*and then we obtain the alternative formula*

(34) $$\begin{array}{c} S_{v}\left(\delta |X\right)=\underset{V\in B_{\delta }\left(X\right)}{\inf}H\left(V\right)\;\;\;\left(\delta \in \left[0,1\right)\right) \end{array}$$

*without an offset term, where*

(35) $$\begin{array}{c} H\left(V\right):=\underset{n\to \infty }{lim sup}\frac{1}{n}H\left(V^{n}\right) \end{array}$$

*is the sup-entropy rate for V with the Shannon entropy $H(V^{n})$. The proof of (34) is given in Appendix B.*

*The same remark also applies to general formulas to be established in the subsequent sections.*

**Remark** **6.** *Independently of this work, Tomita, Uyematsu, and Matsumoto [34] have investigated the following problem: the coin distribution is given by fair coin-tossing and the average number of coin tosses should be asymptotically minimized as in [3] while the variational distance between the target and approximated distributions should satisfy (19). In this case, the asymptotically minimum average number of coin tosses is also characterized by the right-hand side (r.h.s.) of (32) (cf. [34]). Since the coin distribution is restricted to that given by fair coin-tossing with a stopping algorithm, realizations of $L_{n}$ must satisfy the Kraft inequality (for prefix codes), whereas the problem addressed in this paper allows the probability distribution of $L_{n}$ to be an arbitrary discrete one, not necessarily implying prefix codes. In this sense, our problem is more relaxed, while the coin is constrained to be conditionally independent given $L_{n}$. Theorem 3 indicates that the v(δ)-resolvability does not differ in both problems. Later, we shall show that, even in the case where the coin distribution may be any general source X, the δ-resolvability remains the same (cf. Theorem 5 and Remark 14).*

On the other hand, we now define the following information quantity (to be used in Remark 7 below) to discuss the relationship with VL source coding: For δ∈[0,1) we define

(36) $$\begin{array}{c} G_{\left[\delta \right]}\left(X^{n}\right)=\underset{\begin{array}{c} A_{n}\subseteq X^{n}:\\ \mathrm{Pr}\{X^{n}\in A_{n}\}\geq 1-\delta \end{array}}{\inf}\sum_{x\in A_{n}}P_{X^{n}}\left(x\right)\log\frac{1}{P_{X^{n}}\left(x\right)}. \end{array}$$

The $G_{\left[\delta \right]}\left(X^{n}\right)$ is a nonincreasing function of δ. Based on this quantity, we define

(37) $$\begin{array}{cc} G_{\left[\delta \right]}\left(X\right) & =\underset{n\to \infty }{lim sup}\frac{1}{n}G_{\left[\delta \right]}\left(X^{n}\right). \end{array}$$

Then, we have

**Remark** **7.** There is a deep relation between the δ-resolvability problem and VL δ-source coding with the error probability asymptotically not exceeding δ. Koga and Yamamoto [35] (also, cf. Han [36]) showed that the minimum average length rate $R_{v}^{*}\left(\delta |X\right)$ of VL δ-source codes is given by

(38) $$\begin{array}{c} R_{v}^{*}\left(\delta |X\right)=\lim_{\gamma ↓0}G_{\left[\delta +\gamma \right]}\left(X\right)\;\;\;\left(\forall \delta \in \left[0,1\right)\right). \end{array}$$

*Theorem 3 and Proposition 1 (to be shown just below) reveal that*

(39) $$\begin{array}{c} S_{v}\left(\delta |X\right)=R_{v}^{*}\left(\delta |X\right)\;\;\;\left(\forall \delta \in \left[0,1\right)\right). \end{array}$$

The following proposition shows a general relationship between $G_{\left[\delta \right]}\left(X\right)$ and $H_{\left[\delta \right]}\left(X\right)$.

**Proposition** **1.** *For any general source X,*

(40) $$\begin{array}{c} H_{\left[\delta \right]}\left(X\right)=G_{\left[\delta \right]}\left(X\right)\leq \left(1-\delta \right){\bar{H}}_{\delta -\gamma }\left(X\right)\;\;\;\left(\forall \delta \in \left(0,1\right),\forall \gamma \in \left(0,\delta \right]\right). \end{array}$$

*In particular,*

(41) $$\begin{array}{c} \lim_{\gamma ↓0}H_{\left[\delta +\gamma \right]}\left(X\right)=\lim_{\gamma ↓0}G_{\left[\delta +\gamma \right]}\left(X\right)\leq \left(1-\delta \right){\bar{H}}_{\delta }\left(X\right)\;\;\;\left(\forall \delta \in \left[0,1\right)\right). \end{array}$$

(*Proof*) See Appendix C.

By plugging γ=δ into (40), a looser but sometimes useful bound

(42) $$\begin{array}{c} H_{\left[\delta \right]}\left(X\right)=G_{\left[\delta \right]}\left(X\right)\leq \left(1-\delta \right)\bar{H}\left(X\right) \end{array}$$

can be obtained. Equation (40) has been derived by [21], which improves a bound established in [35,37]. In view of Theorems 2 and 3, (41) in Proposition 1 implies

(43) $$\begin{array}{c} S_{v}\left(\delta |X\right)\leq \left(1-\delta \right)S_{f}\left(\delta |X\right) \end{array}$$

for all δ∈[0,1), where $S_{f}\left(\delta |X\right)$ denotes the f(δ)-resolvability. This general relationship elucidates the advantage of the use of VL uniform random numbers to make the average length rate lower. The proposition also claims that $G_{\left[\delta \right]}\left(X\right)$ coincides with $H_{\left[\delta \right]}\left(X\right)$ for all δ∈[0,1) for any general source X.

A consequence of Theorem 3 is the following corollary:

**Corollary** **1.** *Let $X={\left\{X^{n}\right\}}_{n=1}^{\infty }$ be a one-point spectrum source ($\bar{H}\left(X\right)=\underline{H}\left(X\right)$) with $X^{n}=\left(X_{1},X_{2},\ldots ,X_{n}\right)$, then we have*

(44) $$\begin{array}{c} S_{f}\left(\delta |X\right)={\bar{H}}_{\delta }\left(X\right)=H^{*}\left(X\right)\;\;\;\left(\forall \delta \in \left[0,1\right)\right), \end{array}$$

where $H^{*}\left(X\right):=\bar{H}\left(X\right)=\underline{H}\left(X\right)$. Moreover, it holds that

(45) $$\begin{array}{c} S_{v}\left(\delta |X\right)=H_{\left[\delta \right]}\left(X\right)=G_{\left[\delta \right]}\left(X\right)=\left(1-\delta \right)H^{*}\left(X\right)\;\;\;\left(\forall \delta \in \left[0,1\right)\right), \end{array}$$

where we notice that $H^{*}\left(X\right)=H\left(X_{1}\right)$ (entropy) for stationary memoryless sources.

*(Proof) See Appendix D.*

Now, we are ready to give the proof of Theorem 3:

**Proof** **of** **Theorem** **3.**

(1) Converse Part:
  Let *R* be v(δ)-achievable. Then, there exists $U^{\left(L_{n}\right)}$ and $\varphi_{n}$ satisfying (18) and
  (46) $$\begin{array}{cc} \; & \underset{n\to \infty }{lim sup}\;\delta_{n}\leq \delta , \end{array}$$
  where we define $\delta_{n}=d\left(P_{X^{n}},P_{{\tilde{X}}^{n}}\right)$ with ${\tilde{X}}^{n}=\varphi_{n}\left(U^{\left(L_{n}\right)}\right)$. Equation (46) implies that, for any given γ>0, it holds that $\delta_{n}\leq \delta +\gamma$ for all $n\geq n_{0}$ with some $n_{0}>0$, and thus we have
  (47) $$\begin{array}{c} H_{\left[\delta +\gamma \right]}\left(X^{n}\right)\leq H_{\left[\delta_{n}\right]}\left(X^{n}\right)\;\;\;\left(\forall n\geq n_{0}\right), \end{array}$$
  because $H_{\left[\delta \right]}\left(X^{n}\right)$ is a nonincreasing function of δ. Since $P_{{\tilde{X}}^{n}}\in B_{\delta_{n}}\left(X^{n}\right)$, we have
  (48) $$\begin{array}{c} H_{\left[\delta_{n}\right]}\left(X^{n}\right)\leq H\left({\tilde{X}}^{n}\right). \end{array}$$
  On the other hand, it follows from (23) that
  (49) $$\begin{array}{cc} H({\tilde{X}}^{n}) & \leq H\left(U^{\left(L_{n}\right)}\right)=E\left[L_{n}\right]+H\left(L_{n}\right), \end{array}$$
  where the inequality is due to the fact that $\varphi_{n}$ is a deterministic mapping and ${\tilde{X}}^{n}=\varphi_{n}\left(U^{\left(L_{n}\right)}\right)$.
  Combining (47)–(49) yields
  (50) $$\begin{array}{cc} H_{\left[\delta +\gamma \right]}\left(X\right) & =\underset{n\to \infty }{lim sup}\frac{1}{n}H_{\left[\delta +\gamma \right]}\left(X^{n}\right)\\ \; & \leq \underset{n\to \infty }{lim sup}\frac{1}{n}E\left[L_{n}\right]+\underset{n\to \infty }{lim sup}\frac{1}{n}H\left(L_{n}\right)\leq R, \end{array}$$
  where we have used (18) and (25) for the last inequality. Since γ>0 is arbitrary, we obtain
  (51) $$\begin{array}{c} \lim_{\gamma ↓0}H_{\left[\delta +\gamma \right]}\left(X\right)\leq R. \end{array}$$
(2) Direct Part:
  Without loss of generality, we assume that $H^{+}:=\lim_{\gamma ↓0}H_{\left[\delta +\gamma \right]}\left(X\right)$ is finite ($H^{+}<+\infty$). Letting $R=H^{+}+3\gamma$, where γ>0 is an arbitrary constant, we shall show that *R* is v(δ)-achievable. In what follows, we use a simpler form of *information spectrum slicing* [2], where each piece of sliced information quantized to a positive integer *ℓ* is approximated by the uniform random number $U^{\left(\ell \right)}$ of the length *ℓ*.
  First, we note that
  (52) $$\begin{array}{c} H^{+}\geq H_{\left[\delta +\gamma \right]}\left(X\right)\geq \frac{1}{n}H_{\left[\delta +\gamma \right]}\left(X^{n}\right)-\gamma \;\;\;\;\left(\forall n>n_{0}\right) \end{array}$$
  because of the monotonicity of $H_{\left[\delta \right]}\left(X\right)$ in δ. Let $V^{n}$ be a random variable subject to $P_{V^{n}}\in B_{\delta +\gamma }\left(X^{n}\right)$, which satisfies
  (53) $$\begin{array}{c} H_{\left[\delta +\gamma \right]}\left(X^{n}\right)+\gamma \geq H\left(V^{n}\right). \end{array}$$
  For γ>0, we can choose a $c_{n}>0$ so large that
  (54) $$\begin{array}{c} \mathrm{Pr}\{V^{n}\notin T_{n}\}\leq \gamma \end{array}$$
  where
  (55) $$\begin{array}{c} T_{n}:=\left\{x\in X^{n}:\frac{1}{n}\log\frac{1}{P_{V^{n}}\left(x\right)}\leq c_{n}\right\}. \end{array}$$
  We also define
  (56) $$\begin{array}{c} \ell \left(x\right):=\left\{\begin{array}{cc} \left\lceil \log\frac{1}{P_{V^{n}}\left(x\right)}+n\gamma \right\rceil & for\;x\in T_{n}\\ 0 & \mathrm{otherwise}. \end{array}\right. \end{array}$$
  For $m=0,1,\ldots ,\beta_{n}:=\left\lceil n\left(c_{n}+\gamma \right)\right\rceil$, set
  (57) $$\begin{array}{c} S_{n}\left(m\right):=\left\{x\in X^{n}:\ell \left(x\right)=m\right\}, \end{array}$$
  then these sets form a partition of $X^{n}$, i.e.,
  (58) $$\begin{array}{c} \overset{\beta_{n}}{\underset{m=0}{⋃}}S_{n}\left(m\right)=X^{n}\;\;\;and\;\;\;\overset{\beta_{n}}{\underset{m=1}{⋃}}S_{n}\left(m\right)=T_{n}. \end{array}$$
  We set $L_{n}$ so that
  (59) $$\begin{array}{c} \mathrm{Pr}\left\{L_{n}=m\right\}=\mathrm{Pr}\left\{V^{n}\in S_{n}\left(m\right)\right\}, \end{array}$$
  where it is obvious that $\sum_{m=0}^{\beta_{n}}\mathrm{Pr}\left\{L_{n}=m\right\}=1$, and, hence, the probability distribution of the VL uniform random number $U^{\left(L_{n}\right)}$ is given as
  (60) $$\begin{array}{c} P_{U^{\left(L_{n}\right)}}\left(u,m\right):=\mathrm{Pr}\left\{U^{\left(L_{n}\right)}=u,L_{n}=m\right\}=\frac{\mathrm{Pr}\{V^{n}\in S_{n}\left(m\right)\}}{K^{m}}\;\;\left(\forall u\in U^{m}\right). \end{array}$$

(a) Construction of Mapping $\varphi_{n}:U^{*}\to X^{n}$:
  Index the elements in $S_{n}\left(m\right)$ as $x_{1},x_{2},\ldots ,x_{|S_{n}\left(m\right)|}\;\left(m=1,2,\cdots \right)$, where
  (61) $$\begin{array}{c} |S_{n}\left(m\right)|\leq K^{m-n\gamma } \end{array}$$
  since for $x\in S_{n}\left(m\right)$
  (62) $$\begin{array}{c} \log\frac{1}{P_{V^{n}}\left(x\right)}\leq m-n\gamma \;⟺\;P_{V^{n}}\left(x\right)\geq K^{-(m-n\gamma )}, \end{array}$$
  and, therefore,
  (63) $$\begin{array}{c} 1\geq \sum_{x\in S_{n}\left(m\right)}P_{V^{n}}\left(x\right)\geq \sum_{x\in S_{n}\left(m\right)}K^{-(m-n\gamma )}=\left|S_{n}\left(m\right)\right|K^{-(m-n\gamma )}. \end{array}$$
  For $i=1,2,\ldots ,|S_{n}\left(m\right)|$, define ${\tilde{A}}_{i}^{\left(m\right)}\subset U^{m}$ as the set of sequences $u\in U^{m}$ so that
  (64) $$\begin{array}{c} \sum_{u\in {\tilde{A}}_{i}^{\left(m\right)}}P_{U^{\left(L_{n}\right)}}\left(u,m\right)\leq P_{V^{n}}\left(x_{i}\right)<\sum_{u\in {\tilde{A}}_{i}^{\left(m\right)}}P_{U^{\left(L_{n}\right)}}\left(u,m\right)+\frac{\mathrm{Pr}\{V^{n}\in S_{n}\left(m\right)\}}{K^{m}} \end{array}$$
  and
  (65) $$\begin{array}{c} {\tilde{A}}_{i}^{\left(m\right)}\cap {\tilde{A}}_{j}^{\left(m\right)}=\emptyset \;\;\;\;\left(i\neq j\right). \end{array}$$
  If
  (66) $$\begin{array}{c} \sum_{i=1}^{|S_{n}\left(m\right)|}\sum_{u\in {\tilde{A}}_{i}^{\left(m\right)}}P_{U^{\left(L_{n}\right)}}\left(u,m\right)<\sum_{i=1}^{|S_{n}\left(m\right)|}P_{V^{n}}\left(x_{i}\right)=\mathrm{Pr}\left\{V^{n}\in S_{n}\left(m\right)\right\}, \end{array}$$
  then add a $u_{i}\in U^{m}\setminus \left(\cup_{j}{\tilde{A}}_{j}^{\left(m\right)}\right)$ to obtain
  (67) $$\begin{array}{c} A_{i}^{\left(m\right)}={\tilde{A}}_{i}^{\left(m\right)}\cup \left\{u_{i}\right\} \end{array}$$
  for i=1,2,… in order, until it holds that with some $1\leq c\leq |S_{n}\left(m\right)|$
  (68) $$\begin{array}{c} \overset{c}{\underset{i=1}{⋃}}A_{i}^{\left(m\right)}\cup \overset{|S_{n}\left(m\right)|}{\underset{i=c+1}{⋃}}{\tilde{A}}_{i}^{\left(m\right)}=U^{m}, \end{array}$$
  where $u_{1},u_{2},\cdots$ are selected to be all different. Since $|U^{m}|=K^{m}$ and
  (69) $$\begin{array}{c} \sum_{u\in U^{m}}P_{U^{\left(L_{n}\right)}}\left(u,m\right)=\sum_{u\in U^{m}}\frac{\mathrm{Pr}\{V^{n}\in S_{n}\left(m\right)\}}{K^{m}}=\mathrm{Pr}\left\{V^{n}\in S_{n}\left(m\right)\right\}, \end{array}$$
  such a $1\leq c\leq |S_{n}\left(m\right)|$ always exists. For simplicity, we set for $i=c+1,c+2,\ldots ,|S_{n}\left(m\right)|$
  (70) $$\begin{array}{c} A_{i}^{\left(m\right)}={\tilde{A}}_{i}^{\left(m\right)} \end{array}$$
  and for $i=1,2,\ldots ,|S_{n}\left(m\right)|$
  (71) $$\begin{array}{c} \varphi_{n}\left(u\right)=x_{i}\;\;for\;u\in A_{i}^{\left(m\right)}, \end{array}$$
  which defines the random variable ${\tilde{X}}^{n}$ with values in $X^{n}$ such that
  (72) $$\begin{array}{c} P_{{\tilde{X}}^{n}}\left(x_{i}\right)=\sum_{u\in A_{i}^{\left(m\right)}}P_{U^{\left(L_{n}\right)}}\left(u,m\right)\;\;\;\left(x_{i}\in S_{n}\left(m\right)\right), \end{array}$$
  that is, ${\tilde{X}}^{n}=\varphi_{n}\left(U^{\left(L_{n}\right)}\right)$, where if $X^{n}\setminus T_{n}\neq \emptyset$, we choose some $x_{0}\in X^{n}\setminus T_{n}$ and set
  (73) $$\begin{array}{c} P_{{\tilde{X}}^{n}}\left(x_{0}\right)=\mathrm{Pr}\left\{V^{n}\notin T_{n}\right\}\;\;\mathrm{and}\;\;\varphi_{n}\left(\lambda \right)=x_{0}. \end{array}$$
  Notice that, by this construction, we have
  (74) $$\begin{array}{c} |P_{{\tilde{X}}^{n}}\left(x_{i}\right)-P_{V^{n}}\left(x_{i}\right)|\leq \frac{\mathrm{Pr}\{V^{n}\in S_{n}\left(m\right)\}}{K^{m}} \end{array}$$
  for $i=1,2,\ldots ,|S_{n}\left(m\right)|;m=1,2,\ldots ,\beta_{n}$, and
  (75) $$\begin{array}{c} \mathrm{Pr}\left\{{\tilde{X}}^{n}\notin T_{n}\right\}=\mathrm{Pr}\left\{V^{n}\notin T_{n}\right\}\leq \gamma . \end{array}$$
(b) *Evaluation of Average Length:*
  Since m=0 does not contribute to the average length $E[L_{n}]$, it is evaluated as follows:
  (76) $$\begin{array}{cc} E[L_{n}] & =\sum_{m=1}^{\beta_{n}}\sum_{u\in U^{m}}P_{U^{\left(L_{n}\right)}}\left(u,m\right)\cdot m\\ \; & =\sum_{m=1}^{\beta_{n}}\sum_{i=1}^{|S_{n}\left(m\right)|}\sum_{u\in A_{i}^{\left(m\right)}}P_{U^{\left(L_{n}\right)}}\left(u,m\right)\cdot m\\ \; & =\sum_{m=1}^{\beta_{n}}\sum_{x_{i}\in S_{n}\left(m\right)}P_{{\tilde{X}}^{n}}\left(x_{i}\right)\cdot m, \end{array}$$
  where we have used $U^{m}={⋃}_{i=1}^{|S_{n}\left(m\right)|}A_{i}^{\left(m\right)}$ and (72). For $x_{i}\in S_{n}\left(m\right)$, we obtain from (74)
  (77) $$\begin{array}{cc} P_{{\tilde{X}}^{n}}\left(x_{i}\right) & \leq P_{V^{n}}\left(x_{i}\right)+\frac{\mathrm{Pr}\{V^{n}\in S_{n}\left(m\right)\}}{K^{m}}\\ \; & \leq P_{V^{n}}\left(x_{i}\right)\left(1+\frac{1}{P_{V^{n}}\left(x_{i}\right)K^{m}}\right)\\ \; & \leq P_{V^{n}}\left(x_{i}\right)\left(1+\frac{1}{K^{n\gamma }}\right), \end{array}$$
  where, to derive the last inequality, we have used (62). Plugging the inequality
  (78) $$\begin{array}{c} m\leq \log\frac{1}{P_{V^{n}}\left(x_{i}\right)}+n\gamma +1\;\;\left(\forall x_{i}\in S_{n}\left(m\right)\right) \end{array}$$
  and (77) into (76), we obtain
  (79) $$\begin{array}{cc} E[L_{n}] & \leq \left(1+\frac{1}{K^{n\gamma }}\right)\sum_{m=1}^{\beta_{n}}\sum_{x_{i}\in S_{n}\left(m\right)}P_{V^{n}}\left(x_{i}\right)\left(\log\frac{1}{P_{V^{n}}\left(x_{i}\right)}+n\gamma +1\right)\\ \; & \leq \left(1+\frac{1}{K^{n\gamma }}\right)\left(H(V^{n})+n\gamma +1\right), \end{array}$$
  which yields
  (80) $$\begin{array}{cc} \underset{n\to \infty }{lim sup}\frac{1}{n}E\left[L_{n}\right] & \leq \underset{n\to \infty }{lim sup}\frac{1}{n}H\left(V^{n}\right)+2\gamma \\ \; & \leq \underset{n\to \infty }{lim sup}\frac{1}{n}H_{\left[\delta +\gamma \right]}\left(X^{n}\right)+3\gamma \\ \; & =H_{\left[\delta +\gamma \right]}\left(X\right)+3\gamma \\ \; & \leq H^{+}+3\gamma =R, \end{array}$$
  where the second inequality follows from (53) and the last one is due to (52).
(c) *Evaluation of Variational Distance:*
  From (61) and (74), we have
  (81) $$\begin{array}{c} \sum_{x\in S_{n}\left(m\right)}\left|P_{{\tilde{X}}^{n}}\left(x\right)-P_{V^{n}}\left(x\right)\right|\leq \frac{|S_{n}\left(m\right)|\mathrm{Pr}\left\{V^{n}\in S_{n}\left(m\right)\right\}}{K^{m}}\leq \frac{\mathrm{Pr}\{V^{n}\in S_{n}\left(m\right)\}}{K^{n\gamma }}, \end{array}$$
  which, in view of (58), leads to
  (82) $$\begin{array}{cc} d(P_{{\tilde{X}}^{n}},P_{V^{n}}) & =\frac{1}{2}\sum_{x\in T_{n}}|P_{{\tilde{X}}^{n}}\left(x\right)-P_{V^{n}}\left(x\right)|+\frac{1}{2}\sum_{x\notin T_{n}}\left|P_{{\tilde{X}}^{n}}\left(x\right)-P_{V^{n}}\left(x\right)\right|\\ \; & \leq \frac{1}{2}\sum_{m=1}^{\beta_{n}}\sum_{x\in S_{n}\left(m\right)}\left|P_{{\tilde{X}}^{n}}\left(x\right)-P_{V^{n}}\left(x\right)\right|\\ \; & \;\;\;\;\;\;+\frac{1}{2}\left(\mathrm{Pr}\left\{{\tilde{X}}^{n}\notin T_{n}\right\}+\mathrm{Pr}\left\{V^{n}\notin T_{n}\right\}\right)\\ \; & \leq \frac{1}{2}\sum_{m=1}^{\beta_{n}}\frac{\mathrm{Pr}\{V^{n}\in S_{n}\left(m\right)\}}{K^{n\gamma }}+\gamma \leq \frac{1}{2}K^{-n\gamma }+\gamma , \end{array}$$
  where we have used (75) to obtain the leftmost inequality in (82). By the triangle inequality, we obtain
  (83) $$\begin{array}{c} d\left(P_{X^{n}},P_{{\tilde{X}}^{n}}\right)\leq d\left(P_{X^{n}},P_{V^{n}}\right)+d\left(P_{{\tilde{X}}^{n}},P_{V^{n}}\right)\leq \delta +2\gamma +\frac{1}{2}K^{-n\gamma }, \end{array}$$
  where the last inequality follows because $P_{V^{n}}\in B_{\delta +\gamma }\left(X^{n}\right)$. Thus, we obtain from (83)
  (84) $$\begin{array}{cc} \underset{n\to \infty }{lim sup}d\left(P_{X^{n}},P_{{\tilde{X}}^{n}}\right) & \leq \delta +2\gamma . \end{array}$$
  Since γ>0 is arbitrary and we have (80), we conclude that *R* is v(δ)-achievable.

□

### 3.4. General Formula for δ=0

In this subsection, we consider the special case with δ=0. In this case, we can elucidate the relationship between the minimum achievable rates for VL source codes with an asymptotically vanishing decoding error probability and the FL source codes.

We obtain the following corollary from Theorem 3 and Proposition 1:

**Corollary** **2.** *For any general target source X,*

(85) $$\begin{array}{c} S_{v}\left(X\right)=\lim_{\gamma ↓0}G_{\left[\gamma \right]}\left(X\right), \end{array}$$

*where $G_{\left[\gamma \right]}\left(X\right)$ is defined in (37).*

It has been shown by Han [2] that any source $X={\left\{X^{n}\right\}}_{n=1}^{\infty }$ satisfying the *uniform integrability* (cf. Han [2]) satisfies

(86) $$\begin{array}{c} \lim_{\gamma ↓0}G_{\left[\gamma \right]}\left(X\right)=H\left(X\right):=\underset{n\to \infty }{lim sup}\frac{1}{n}H\left(X^{n}\right), \end{array}$$

where H(X) is called the *sup-entropy rate*. Notice here, in particular, that the finiteness of an alphabet implies the uniform integrability [2]. Thus, we obtain the following corollary:

**Corollary** **3.** *For any finite alphabet target source X,*

(87) $$\begin{array}{c} S_{v}\left(X\right)=H\left(X\right). \end{array}$$

**Remark** **8.** As in the case of FL resolvability and FL source coding problems, $S_{v}\left(X\right)$ is tightly related to VL source codes with vanishing decoding error probabilities. Denoting by $R_{v}^{*}\left(X\right)$ the minimum error-vanishing VL -achievable rate for a source X, Han [36] has shown that

(88) $$\begin{array}{c} R_{v}^{*}\left(X\right)=\lim_{\gamma ↓0}G_{\left[\gamma \right]}\left(X\right), \end{array}$$

*and, hence, from Corollary 3, it is concluded that*

(89) $$\begin{array}{c} S_{v}\left(X\right)=R_{v}^{*}\left(X\right). \end{array}$$

*In addition, if a general source*X *satisfies the uniform integrability and the strong converse property (cf. Han [2]), then equation (86) holds and hence it follows from ([2], Theorem 1.7.1) that*

(90) $$\begin{array}{c} S_{f}\left(X\right)=S_{v}\left(X\right)=R_{v}^{*}\left(X\right)=R_{v}\left(X\right)=R_{f}\left(X\right)=H\left(X\right), \end{array}$$

*where $R_{f}\left(X\right):=R_{f}\left(0|X\right)$ and $R_{v}\left(X\right)$ denotes the minimum achievable rate of VL source codes with zero error probabilities for all n=1,2,⋯.*

**Remark** **9.** *Han and Verdú [6] have discussed the problem of mean-resolvability for the target distribution $P_{X^{n}}$. In this problem, the coin distribution may be a general source $\tilde{X}={\left\{{\tilde{X}}^{n}\right\}}_{n=1}^{\infty }$, where ${\tilde{X}}^{n}$ is a random variable that takes values in $X^{n}$ with the average length rate $\frac{1}{n}E\left[L_{n}\right]$ in (18) replaced with the entropy rate $\frac{1}{n}H\left({\tilde{X}}^{n}\right)$. Denoting by ${\bar{S}}_{v}\left(X\right)$ the mean-resolvability, which is defined as the infimum of v-achievable rates for a general source X (with the countably infinite alphabet), we can easily verify that any mean-resolution rate $R>{\bar{S}}_{v}\left(X\right)$ must satisfy $R\geq \lim_{\gamma ↓0}G_{\left[\gamma \right]}\left(X\right)$ so that $S_{v}\left(X\right)\leq {\bar{S}}_{v}\left(X\right)$. On the other hand, $S_{v}\left(X\right)\geq {\bar{S}}_{v}\left(X\right)$ by definition. Thus, in view of Corollary 2, we have*

**Corollary** **4.** *For any general target source X,*

(91) $$\begin{array}{c} S_{v}\left(X\right)={\bar{S}}_{v}\left(X\right)=\lim_{\gamma ↓0}G_{\left[\gamma \right]}\left(X\right). \end{array}$$

## 4. VL Resolvability: Divergence

So far, we have considered the problem of VL resolvability, in which the approximation level is measured by the variational distance between $X^{n}$ and ${\tilde{X}}^{n}$. It is sometimes of use to deal with another quantity as an approximation measure. In this section, we use the (unnormalized) divergence as the approximation measure.

### 4.1. Definitions

In this subsection, we address the following problem.

**Definition** **4** (VL δ-resolvability: divergence)**.** A resolution rate R≥0 is said to be VL δ-achievable or simply $v_{D}\left(\delta \right)$-achievable (under the divergence) with δ≥0, if there exists a VL uniform random number $U^{\left(L_{n}\right)}$ and a deterministic mapping $\varphi_{n}:U^{*}\to X^{n}$ satisfying

(92) $$\begin{array}{cc} \underset{n\to \infty }{lim sup}\frac{1}{n}E\left[L_{n}\right] & \leq R, \end{array}$$

(93) $$\begin{array}{cc} \underset{n\to \infty }{lim sup}D({\tilde{X}}^{n}\left|\right|X^{n}) & \leq \delta , \end{array}$$

*where ${\tilde{X}}^{n}=\varphi_{n}\left(U^{\left(L_{n}\right)}\right)$ and $D({\tilde{X}}^{n}||X^{n})$ denotes the divergence between $P_{{\tilde{X}}^{n}}$ and $P_{X^{n}}$:*

(94) $$\begin{array}{c} D({\tilde{X}}^{n}\left|\right|X^{n})=\sum_{x\in X^{n}}P_{{\tilde{X}}^{n}}\left(x\right)\log\frac{P_{{\tilde{X}}^{n}}\left(x\right)}{P_{X^{n}}\left(x\right)}. \end{array}$$

*The infimum of all $v_{D}\left(\delta \right)$-achievable rates, i.e.,*

(95) $$\begin{array}{c} S_{v}^{D}\left(\delta |X\right):=\inf\left\{R:\;R\;is\;v_{D}\left(\delta \right)-achievable\right\} \end{array}$$

*is called the VL* δ*-resolvability or simply the $v_{D}\left(\delta \right)$-resolvability.*

**Remark** **10.** *The measure of approximation is now the divergence $D({\tilde{X}}^{n}||X^{n})$ but not its reversed version $D(X^{n}||{\tilde{X}}^{n})$. In the context of resolvability, divergence $D({\tilde{X}}^{n}||X^{n})$ (and its counterpart in the case of channel resolvability) is usually employed as in [6,7,9]. We also use this type of divergence in the subsequent sections.*

To establish the general formula for $S_{v}^{D}\left(\delta |X\right)$, we introduce the following quantity for a general source $X={\left\{X^{n}\right\}}_{n=1}^{\infty }$. Recall that $P(X^{n})$ denotes the set of all probability distributions on $X^{n}$. For δ≥0, defining the *δ-ball* using the divergence as

(96) $$\begin{array}{c} B_{\delta }^{D}\left(X^{n}\right)=\left\{P_{V^{n}}\in P\left(X^{n}\right):D(V^{n}\left|\right|X^{n})\leq \delta \right\}, \end{array}$$

we introduce the following quantity, referred to as the *smooth entropy* using the divergence:

(97) $$\begin{array}{cc} H_{\left[\delta \right]}^{D}\left(X^{n}\right) & :=\underset{P_{V^{n}}\in B_{\delta }^{D}\left(X^{n}\right)}{\inf}H\left(V^{n}\right), \end{array}$$

where $H(V^{n})$ denotes the Shannon entropy of $P_{V^{n}}$. Obviously, $H_{\left[\delta \right]}^{D}\left(X^{n}\right)$ is a nonincreasing function of δ. Now, we define

(98) $$\begin{array}{cc} H_{\left[\delta \right]}^{D}\left(X\right) & =\underset{n\to \infty }{lim sup}\frac{1}{n}H_{\left[\delta \right]}^{D}\left(X^{n}\right). \end{array}$$

The following lemma is used to derive Corollary 5 of Theorem 4 below in the next subsection.

**Lemma** **1.** *For any general source X,*

(99) $$\begin{array}{c} H_{\left[\delta \right]}\left(X\right)\leq H_{\left[g(\delta )\right]}^{D}\left(X\right)\;\;\;\left(\delta \;\geq 0\right), \end{array}$$

*where we define $g\left(\delta \right)=2\delta^{2}/\ln K$, and*

(100) $$\begin{array}{c} \lim_{\delta ↓0}G_{\left[\delta \right]}\left(X\right)=\lim_{\delta ↓0}H_{\left[\delta \right]}\left(X\right)=\lim_{\delta ↓0}H_{\left[\delta \right]}^{D}\left(X\right)\leq \bar{H}\left(X\right). \end{array}$$

*(Proof) See Appendix E.*

### 4.2. General Formula

Here, we establish another main theorem, which characterizes $S_{v}^{D}\left(\delta |X\right)$ for all δ≥0 in terms of the smooth entropy using the divergence.

**Theorem** **4.** *For any general target source X,*

(101) $$\begin{array}{c} S_{v}^{D}\left(\delta |X\right)=\lim_{\gamma ↓0}H_{\left[\delta +\gamma \right]}^{D}\left(X\right)\;\;\;\left(\delta \;\geq 0\right). \end{array}$$

**Remark** **11.** *It should be noticed that the approximation measure considered here is not the normalized divergence*

(102) $$\begin{array}{c} \frac{1}{n}D(\varphi_{n}\left(U^{\left(L_{n}\right)}\right)\left|\right|X^{n}), \end{array}$$

*which has been used in the problem of FL δ-resolvability [7]. The achievability scheme given in the proof of the direct part of Theorem 4 can also be used in the case of this relaxed measure. Indeed, denoting the VL δ-resolvability with the normalized divergence by ${\tilde{S}}_{v}^{D}\left(\delta |X\right)$, the general formula for ${\tilde{S}}_{v}^{D}\left(\delta |X\right)$ is given in the same form as (101), if the radius of the δ-ball $B_{\delta }^{D}\left(X^{n}\right)$ in the definition of $H_{\left[\delta \right]}^{D}\left(X^{n}\right)$ is replaced with the normalized divergence. It generally holds that $S_{v}^{D}\left(\delta |X\right)\geq {\tilde{S}}_{v}^{D}\left(\delta |X\right)$ for all δ≥0 because the normalized divergence is smaller than the unnormalized divergence.*

As we have seen in Lemma 1, we generally have $S_{v}^{D}\left(g\left(\delta \right)|X\right)\geq S_{v}\left(\delta |X\right)$ for any δ∈[0,1) with $g\left(\delta \right)=2\delta^{2}/\ln K$. In particular, in the case that δ=0, we obtain the following corollary of Theorems 3 and 4.

**Corollary** **5.** *For any general target source X,*

(103) $$\begin{array}{cc} S_{v}^{D}\left(0|X\right) & =S_{v}\left(X\right). \end{array}$$

Corollary 5 indicates that the $v_{D}\left(0\right)$-resolvability $S_{v}^{D}\left(0|X\right)$ coincides with the v-resolvability $S_{v}\left(X\right)$ and is also characterized by the r.h.s. of (85). By (88), it also implies that $S_{v}^{D}\left(0|X\right)=R_{v}^{*}\left(X\right)$, where $R_{v}^{*}\left(X\right)$ denotes the minimum error-vanishing achievable rate with VL source codes for X.

**Proof** **of** **Theorem** **4.**

(1) Converse Part:
  Let *R* be $v_{D}\left(\delta \right)$-achievable. Then, there exists $U^{\left(L_{n}\right)}$ and $\varphi_{n}$ satisfying (92) and
  (104) $$\begin{array}{cc} \; & \underset{n\to \infty }{lim sup}\delta_{n}\leq \delta , \end{array}$$
  where we define $\delta_{n}=D({\tilde{X}}^{n}\left|\right|X^{n})$ with ${\tilde{X}}^{n}=\varphi_{n}\left(U^{\left(L_{n}\right)}\right)$. Equation (104) implies that, for any given γ>0, it holds that $\delta_{n}\leq \delta +\gamma$ for all $n\geq n_{0}$ with some $n_{0}>0$, and therefore
  (105) $$\begin{array}{c} H_{\left[\delta +\gamma \right]}^{D}\left(X^{n}\right)\leq H_{\left[\delta_{n}\right]}^{D}\left(X^{n}\right)\;\;\;\left(\forall n\geq n_{0}\right) \end{array}$$
  since $H_{\left[\delta \right]}^{D}\left(X^{n}\right)$ is a nonincreasing function of δ. Since $P_{{\tilde{X}}^{n}}\in B_{\delta_{n}}^{D}\left(X^{n}\right)$, we have
  (106) $$\begin{array}{c} H_{\left[\delta_{n}\right]}^{D}\left(X^{n}\right)\leq H\left({\tilde{X}}^{n}\right). \end{array}$$
  On the other hand, it follows from (23) that
  (107) $$\begin{array}{cc} H({\tilde{X}}^{n}) & \leq H\left(U^{\left(L_{n}\right)}\right)=E\left[L_{n}\right]+H\left(L_{n}\right), \end{array}$$
  where the inequality is due to the fact that $\varphi_{n}$ is a deterministic mapping and ${\tilde{X}}^{n}=\varphi_{n}\left(U^{\left(L_{n}\right)}\right)$.
  Combining (105)–(107) yields
  (108) $$\begin{array}{cc} H_{\left[\delta +\gamma \right]}^{D}\left(X\right) & =\underset{n\to \infty }{lim sup}\frac{1}{n}H_{\left[\delta +\gamma \right]}^{D}\left(X^{n}\right)\\ \; & \leq \underset{n\to \infty }{lim sup}\frac{1}{n}E\left[L_{n}\right]+\underset{n\to \infty }{lim sup}\frac{1}{n}H\left(L_{n}\right)\leq R, \end{array}$$
  where we used (25) and (92) for the last inequality. Since γ>0 is arbitrary, we have
  (109) $$\begin{array}{c} \lim_{\gamma ↓0}H_{\left[\delta +\gamma \right]}^{D}\left(X\right)\leq R. \end{array}$$
(2) Direct Part:
  We modify the achievability scheme in the proof of the direct part of Theorem 3. Although the proof of this part is quite similar to that of the direct part of Theorem 3, we give here the full proof in order to avoid subtle possible confusions. We may assume that $H^{+}:=\lim_{\gamma ↓0}H_{\left[\delta +\gamma \right]}^{D}\left(X\right)$ is finite ($H^{+}<+\infty$). Letting $R=H^{+}+\mu$, where μ>0 is an arbitrary constant, we shall show that *R* is $v_{D}\left(\delta \right)$-achievable.
  Let $V^{n}$ be a random variable subject to $P_{V^{n}}\in B_{\delta +\gamma }^{D}\left(X^{n}\right)$ satisfying
  (110) $$\begin{array}{c} H_{\left[\delta +\gamma \right]}^{D}\left(X^{n}\right)+\gamma \geq H\left(V^{n}\right) \end{array}$$
  for any fixed $\gamma \in (0,\frac{1}{2}]$. We can choose a $c_{n}>0$ so large that
  (111) $$\begin{array}{c} \gamma_{0}:=\mathrm{Pr}\left\{V^{n}\notin T_{n}\right\}\leq \gamma \end{array}$$
  where
  (112) $$\begin{array}{c} T_{n}:=\left\{x\in X^{n}:\frac{1}{n}\log\frac{1}{P_{V^{n}}\left(x\right)}\leq c_{n}\right\}. \end{array}$$
  We also define
  (113) $$\begin{array}{c} \ell \left(x\right):=\left\lceil \log\frac{1}{P_{V^{n}}\left(x\right)}+n\gamma \right\rceil \;\;\;for\;x\in T_{n}. \end{array}$$
  Letting, for $m=1,2,\ldots ,\beta_{n}:=\left\lceil n\left(c_{n}+\gamma \right)\right\rceil$,
  (114) $$\begin{array}{c} S_{n}\left(m\right):=\left\{x\in X^{n}:\ell \left(x\right)=m\right\}, \end{array}$$
  these sets form a partition of $T_{n}$:
  (115) $$\begin{array}{c} \overset{\beta_{n}}{\underset{m=1}{⋃}}S_{n}\left(m\right)=T_{n}. \end{array}$$
  We set $L_{n}$ so that
  (116) $$\begin{array}{c} \mathrm{Pr}\left\{L_{n}=m\right\}=\frac{\mathrm{Pr}\{V^{n}\in S_{n}\left(m\right)\}}{\mathrm{Pr}\{V^{n}\in T_{n}\}}=\frac{\mathrm{Pr}\{V^{n}\in S_{n}\left(m\right)\}}{1-\gamma_{0}}, \end{array}$$
  which satisfies
  (117) $$\begin{array}{c} \sum_{m=1}^{\beta_{n}}\mathrm{Pr}\left\{L_{n}=m\right\}=\frac{\mathrm{Pr}\{V^{n}\in T_{n}\}}{1-\gamma_{0}}=1, \end{array}$$
  and, hence, the probability distribution of $U^{\left(L_{n}\right)}$ is given as
  (118) $$\begin{array}{c} P_{U^{\left(L_{n}\right)}}\left(u,m\right):=\mathrm{Pr}\left\{U^{\left(L_{n}\right)}=u,L_{n}=m\right\}=\frac{\mathrm{Pr}\{V^{n}\in S_{n}\left(m\right)\}}{\left(1-\gamma_{0}\right)K^{m}}\;\;\left(\forall u\in U^{m}\right). \end{array}$$

(a) Construction of Mapping $\varphi_{n}:U^{*}\to X^{n}$:
  Index the elements in $S_{n}\left(m\right)$ as $x_{1},x_{2},\ldots ,x_{|S_{n}\left(m\right)|}\;\left(m=1,2,\ldots ,\beta_{n}\right)$, where it holds that
  (119) $$\begin{array}{c} |S_{n}\left(m\right)|\leq K^{m-n\gamma } \end{array}$$
  (cf. (61)–(63)). For $i=1,2,\ldots ,|S_{n}\left(m\right)|$, define ${\tilde{A}}_{i}^{\left(m\right)}\subset U^{m}$ as the set of sequences $u\in U^{m}$ so that
  (120) $$\begin{array}{c} \sum_{u\in {\tilde{A}}_{i}^{\left(m\right)}}P_{U^{\left(L_{n}\right)}}\left(u,m\right)\leq \frac{P_{V^{n}}\left(x_{i}\right)}{1-\gamma_{0}}<\sum_{u\in {\tilde{A}}_{i}^{\left(m\right)}}P_{U^{\left(L_{n}\right)}}\left(u,m\right)+\frac{\mathrm{Pr}\{L_{n}=m\}}{K^{m}} \end{array}$$
  and
  (121) $$\begin{array}{c} {\tilde{A}}_{i}^{\left(m\right)}\cap {\tilde{A}}_{j}^{\left(m\right)}=\emptyset \;\;\;\;\left(i\neq j\right). \end{array}$$
  If
  (122) $$\begin{array}{c} \sum_{i=1}^{|S_{n}\left(m\right)|}\sum_{u\in {\tilde{A}}_{i}^{\left(m\right)}}P_{U^{\left(L_{n}\right)}}\left(u,m\right)<\frac{1}{1-\gamma_{0}}\sum_{i=1}^{|S_{n}\left(m\right)|}P_{V^{n}}\left(x_{i}\right)=\mathrm{Pr}\left\{L_{n}=m\right\}, \end{array}$$
  then add a $u_{i}\in U^{m}\setminus \left(\cup_{j}{\tilde{A}}_{j}^{\left(m\right)}\right)$ to obtain
  (123) $$\begin{array}{c} A_{i}^{\left(m\right)}={\tilde{A}}_{i}^{\left(m\right)}\cup \left\{u_{i}\right\} \end{array}$$
  for i=1,2,… in order, until it holds that with some $1\leq c\leq |S_{n}\left(m\right)|$
  (124) $$\begin{array}{c} \overset{c}{\underset{i=1}{⋃}}A_{i}^{\left(m\right)}\cup \overset{|S_{n}\left(m\right)|}{\underset{i=c+1}{⋃}}{\tilde{A}}_{i}^{\left(m\right)}=U^{m}, \end{array}$$
  where $u_{1},u_{2},\cdots$ are selected to be all distinct. Since $|U^{m}|=K^{m}$ and
  (125) $$\begin{array}{c} \sum_{u\in U^{m}}P_{U^{\left(L_{n}\right)}}\left(u,m\right)=\sum_{u\in U^{m}}\frac{\mathrm{Pr}\{V^{n}\in S_{n}\left(m\right)\}}{\left(1-\gamma_{0}\right)K^{m}}=\mathrm{Pr}\left\{L_{n}=m\right\}, \end{array}$$
  such a $1\leq c\leq |S_{n}\left(m\right)|$ always exists. For simplicity, we set for $i=c+1,c+2,\ldots ,|S_{n}\left(m\right)|$
  (126) $$\begin{array}{c} A_{i}^{\left(m\right)}={\tilde{A}}_{i}^{\left(m\right)} \end{array}$$
  and for $i=1,2,\ldots ,|S_{n}\left(m\right)|$
  (127) $$\begin{array}{c} \varphi_{n}\left(u\right)=x_{i}\;\;for\;u\in A_{i}^{\left(m\right)}, \end{array}$$
  which defines the random variable ${\tilde{X}}^{n}$ with values in $X^{n}$ such that
  (128) $$\begin{array}{c} P_{{\tilde{X}}^{n}}\left(x_{i}\right)=\sum_{u\in A_{i}^{\left(m\right)}}P_{U^{\left(L_{n}\right)}}\left(u,m\right)\;\;\;\left(x_{i}\in S_{n}\left(m\right)\right), \end{array}$$
  that is, ${\tilde{X}}^{n}=\varphi_{n}\left(U^{\left(L_{n}\right)}\right)$. Notice that, by this construction, we have
  (129) $$\begin{array}{c} \left|P_{{\tilde{X}}^{n}}\left(x_{i}\right)-\frac{P_{V^{n}}\left(x_{i}\right)}{1-\gamma_{0}}\right|\leq \frac{\mathrm{Pr}\{L_{n}=m\}}{K^{m}}=\frac{\mathrm{Pr}\{V^{n}\in S_{n}\left(m\right)\}}{\left(1-\gamma_{0}\right)K^{m}} \end{array}$$
  for $i=1,2,\ldots ,|S_{n}\left(m\right)|;m=1,2,\ldots ,\beta_{n}$, and
  (130) $$\begin{array}{c} \mathrm{Pr}\left\{{\tilde{X}}^{n}\notin T_{n}\right\}=0\;\;\;\mathrm{and}\;\;\;\mathrm{Pr}\left\{V^{n}\notin T_{n}\right\}\leq \gamma . \end{array}$$
(b) *Evaluation of Average Length:*
  The average length $E[L_{n}]$ is evaluated as follows:
  (131) $$\begin{array}{cc} E[L_{n}] & =\sum_{m=1}^{\beta_{n}}\sum_{u\in U^{m}}P_{U^{\left(L_{n}\right)}}\left(u,m\right)\cdot m\\ \; & =\sum_{m=1}^{\beta_{n}}\sum_{i=1}^{|S_{n}\left(m\right)|}\sum_{u\in A_{i}^{\left(m\right)}}P_{U^{\left(L_{n}\right)}}\left(u,m\right)\cdot m\\ \; & =\sum_{m=1}^{\beta_{n}}\sum_{x_{i}\in S_{n}\left(m\right)}P_{{\tilde{X}}^{n}}\left(x_{i}\right)\cdot m, \end{array}$$
  where we have used $U^{m}={⋃}_{i=1}^{|S_{n}\left(m\right)|}A_{i}^{\left(m\right)}$ and (128). For $x_{i}\in S_{n}\left(m\right)$ we obtain from (129) and the right inequality of (130)
  (132) $$\begin{array}{cc} P_{{\tilde{X}}^{n}}\left(x_{i}\right) & \leq \frac{P_{V^{n}}\left(x_{i}\right)}{1-\gamma_{0}}+\frac{\mathrm{Pr}\{V^{n}\in S_{n}\left(m\right)\}}{\left(1-\gamma_{0}\right)K^{m}}\\ \; & =\frac{1}{1-\gamma_{0}}\left(P_{V^{n}}\left(x_{i}\right)+\frac{\mathrm{Pr}\{V^{n}\in S_{n}\left(m\right)\}}{K^{m}}\right)\\ \; & \leq \frac{1}{1-\gamma_{0}}\left(1+\frac{1}{P_{V^{n}}\left(x_{i}\right)K^{m}}\right)P_{V^{n}}\left(x_{i}\right)\\ \; & \leq \left(1+2\gamma \right)\left(1+\frac{1}{K^{n\gamma }}\right)P_{V^{n}}\left(x_{i}\right), \end{array}$$
  where, to derive the last inequality, we have used the fact $0\leq \;\gamma_{0}\leq \gamma \leq \frac{1}{2}$ and
  (133) $$\begin{array}{c} P_{V^{n}}\left(x_{i}\right)\geq K^{-(m-n\gamma )}\;\;\;\left(\forall x_{i}\in S_{n}\left(m\right)\right). \end{array}$$
  It should be noticed that (132) also implies that
  (134) $$\begin{array}{c} P_{{\tilde{X}}^{n}}\left(x\right)\leq \left(1+2\gamma \right)\left(1+\frac{1}{K^{n\gamma }}\right)P_{V^{n}}\left(x\right)\;\;\;\left(\forall x\in X^{n}\right) \end{array}$$
  since $P_{{\tilde{X}}^{n}}\left(x\right)=0$ for $x\notin T_{n}={⋃}_{m=1}^{\beta_{n}}S_{n}\left(m\right)$. Plugging the inequality
  (135) $$\begin{array}{c} m\leq \log\frac{1}{P_{V^{n}}\left(x_{i}\right)}+n\gamma +1\;\;\left(\forall x_{i}\in S_{n}\left(m\right)\right) \end{array}$$
  and (132) into (131), we obtain
  (136) $$\begin{array}{cc} E[L_{n}] & \leq \left(1+2\gamma \right)\left(1+\frac{1}{K^{n\gamma }}\right)\\ \; & \;\;\;\;\;\cdot \sum_{m=1}^{\beta_{n}}\sum_{x_{i}\in S_{n}\left(m\right)}P_{V^{n}}\left(x_{i}\right)\left(\log\frac{1}{P_{V^{n}}\left(x_{i}\right)}+n\gamma +1\right)\\ \; & \;\;\;\leq \left(1+2\gamma \right)\left(1+\frac{1}{K^{n\gamma }}\right)\left(H(V^{n})+n\gamma +1\right). \end{array}$$
  Thus, we obtain from (136)
  (137) $$\begin{array}{cc} \underset{n\to \infty }{lim sup}\frac{1}{n}E\left[L_{n}\right] & \leq \left(1+2\gamma \right)\left\{\underset{n\to \infty }{lim sup}\frac{1}{n}H\left(V^{n}\right)+\gamma \right\}\\ \; & \leq \left(1+2\gamma \right)\left\{\underset{n\to \infty }{lim sup}\frac{1}{n}H_{\left[\delta +\gamma \right]}^{D}\left(X^{n}\right)+2\gamma \right\}\\ \; & \leq \left(1+2\gamma \right)\left(H^{+}+2\gamma \right), \end{array}$$
  where the second inequality follows from (110). Since we have assumed that $H^{+}$ is finite and $\gamma \in (0,\frac{1}{2}]$ is arbitrary, the r.h.s. of (137) can be made as close to $H^{+}$ as desired. Therefore, for all sufficiently small γ>0, we obtain
  (138) $$\begin{array}{cc} \underset{n\to \infty }{lim sup}\frac{1}{n}E\left[L_{n}\right] & \leq H^{+}+\mu =R \end{array}$$
(c) *Evaluation of Divergence:*
  The divergence $D({\tilde{X}}^{n}||X^{n})$ can be rewritten as
  (139) $$\begin{array}{c} D({\tilde{X}}^{n}\left|\right|X^{n})=D({\tilde{X}}^{n}\left|\right|V^{n})+E\left[\log\frac{P_{V^{n}}\left({\tilde{X}}^{n}\right)}{P_{X^{n}}\left({\tilde{X}}^{n}\right)}\right]. \end{array}$$
  In view of (132), we obtain
  (140) $$\begin{array}{cc} D({\tilde{X}}^{n}||V^{n}) & =\sum_{m=1}^{\beta_{n}}\sum_{x\in S_{n}\left(m\right)}P_{{\tilde{X}}^{n}}\left(x\right)\log\frac{P_{{\tilde{X}}^{n}}\left(x\right)}{P_{V^{n}}\left(x\right)}\\ \; & \leq \sum_{m=1}^{\beta_{n}}\sum_{x\in S_{n}\left(m\right)}P_{{\tilde{X}}^{n}}\left(x\right)\log\left\{\left(1+2\gamma \right)\left(1+\frac{1}{K^{n\gamma }}\right)\right\}\\ \; & \leq \frac{2\gamma }{\ln K}\;+\log\left(1+\frac{1}{K^{n\gamma }}\right) \end{array}$$
  and
  (141) $$\begin{array}{cc} E\left[\log\frac{P_{V^{n}}\left({\tilde{X}}^{n}\right)}{P_{X^{n}}\left({\tilde{X}}^{n}\right)}\right]\; & =\sum_{x\in X^{n}}P_{{\tilde{X}}^{n}}\left(x\right)\log\frac{P_{V^{n}}\left(x\right)}{P_{X^{n}}\left(x\right)}\\ \; & \leq \left(1+2\gamma \right)\left(1+\frac{1}{K^{n\gamma }}\right)D(V^{n}\left|\right|X^{n})\\ \; & \leq \left(1+2\gamma \right)\left(\delta +\gamma \right)\left(1+\frac{1}{K^{n\gamma }}\right), \end{array}$$
  where to obtain the last inequality we used the fact that $P_{V^{n}}\in B_{\delta +\gamma }^{D}\left(X^{n}\right)$. Plugging (140) and (141) into (139) yields
  (142) $$\begin{array}{cc} \underset{n\to \infty }{lim sup}D({\tilde{X}}^{n}\left|\right|X^{n}) & \leq \frac{2\gamma }{\ln K}+\left(1+2\gamma \right)\left(\delta +\gamma \right)\\ \; & \leq \delta +\gamma (2\delta +5), \end{array}$$
  where we have used the fact that $\frac{2\gamma }{\ln K}\leq 3\gamma$ for all K≥2 and the assumption $0<\gamma \leq \frac{1}{2}$ to derive the last inequality. Since $\gamma \in (0,\frac{1}{2}]$ is arbitrary and we have (138), *R* is $v_{D}\left(\delta \right)$-achievable.

□

## 5. Mean and VL Channel Resolvability

So far, we have studied the problem of *source resolvability*, whereas the problem of *channel resolvability* has been introduced by Han and Verdú [6] to investigate the capacity of identification codes [11]. In a conventional problem of this kind, a target *output distribution* $P_{Y^{n}}$ via a channel $W^{n}$ due to an input $X^{n}$ is approximated by encoding the FL uniform random number $U_{M_{n}}$ as a channel input. In this section, we generalize the problem of such channel resolvability to that in the variable-length setting.

### 5.1. Definitions

Let X and Y be finite or countably infinite alphabets. Let $W={\left\{W^{n}\right\}}_{n=1}^{\infty }$ be a general channel, where $W^{n}:X^{n}\to Y^{n}$ denotes a stochastic mapping. We denote by $Y={\left\{Y^{n}\right\}}_{n=1}^{\infty }$ the output process via W due to an input process $X={\left\{X^{n}\right\}}_{n=1}^{\infty }$, where $X^{n}$ and $Y^{n}$ take values in $X^{n}$ and $Y^{n}$, respectively. Again, we do not impose any assumptions such as stationarity or ergodicity on either X or W. As in the previous sections, we will identify $X^{n}$ and $Y^{n}$ with their probability distributions $P_{X^{n}}$ and $P_{Y^{n}}$, respectively, and these symbols are used interchangeably.

In this section, we consider several types of problems of approximating a target output distribution $P_{Y^{n}}$. The first one is the problem of *mean*-resolvability [6], in which the channel input is allowed to be an arbitrary general source.

**Definition** **5** (mean δ-channel resolvability: variational distance)**.** *Let δ∈[0,1) be fixed arbitrarily. A resolution rate R≥0 is said to be mean δ-achievable for X (under the variational distance) if there exists a general source $\tilde{X}={\left\{{\tilde{X}}^{n}\right\}}_{n=1}^{\infty }$ satisfying*

(143) $$\begin{array}{cc} \underset{n\to \infty }{lim sup}\frac{1}{n}H\left({\tilde{X}}^{n}\right) & \leq R, \end{array}$$

(144) $$\begin{array}{cc} \underset{n\to \infty }{lim sup}d\left(P_{Y^{n}},P_{{\tilde{Y}}^{n}}\right) & \leq \delta , \end{array}$$

*where ${\tilde{Y}}^{n}$ denotes the output via $W^{n}$ due to input ${\tilde{X}}^{n}$. The infimum of all mean δ-achievable rates for X, i.e.,*

(145) $$\begin{array}{c} {\bar{S}}_{v}\left(\delta |X,W\right):=\inf\left\{R:\;R\;is\;mean\;\delta -achievable\;for\;X\right\} \end{array}$$

*is referred to as the mean δ-resolvability for X. We also define the mean δ-resolvability for the worst input as*

(146) $$\begin{array}{c} {\bar{S}}_{v}\left(\delta |W\right):=\underset{X}{\sup}{\bar{S}}_{v}\left(\delta |X,W\right). \end{array}$$

On the other hand, we may also consider the problem of VL channel resolvability. Here, the VL uniform random number $U^{\left(L_{n}\right)}$ is defined as in the foregoing sections. Consider the problem of approximating the target output distribution $P_{Y^{n}}$ via $W^{n}$ due to $X^{n}$ by using another input ${\tilde{X}}^{n}=\varphi_{n}\left(U^{\left(L_{n}\right)}\right)$ with a deterministic mapping $\varphi_{n}:U^{*}\to X^{n}$.

**Definition** **6** (VL δ-channel resolvability: variational distance)**.** Let δ∈[0,1) be fixed arbitrarily. A resolution rate R≥0 is said to be VL δ-achievable or simply v(δ)-achievable for X (under the variational distance) if there exists a VL uniform random number $U^{\left(L_{n}\right)}$ and a deterministic mapping $\varphi_{n}:U^{*}\to X^{n}$ satisfying

(147) $$\begin{array}{cc} \underset{n\to \infty }{lim sup}\frac{1}{n}E\left[L_{n}\right] & \leq R, \end{array}$$

(148) $$\begin{array}{cc} \underset{n\to \infty }{lim sup}d\left(P_{Y^{n}},P_{{\tilde{Y}}^{n}}\right) & \leq \delta , \end{array}$$

*where E[·] denotes the expected value and ${\tilde{Y}}^{n}$ denotes the output via $W^{n}$ due to input ${\tilde{X}}^{n}=\varphi_{n}\left(U^{\left(L_{n}\right)}\right)$. The infimum of all v(δ)-achievable rates for X, i.e.,*

(149) $$\begin{array}{c} S_{v}\left(\delta |X,W\right):=\inf\left\{R:\;R\;is\;v(\delta )-achievable\;for\;X\right\} \end{array}$$

*is called the VL δ-channel resolvability or simply v(δ)-channel resolvability for X. We also define the VL δ-channel resolvability or simply v(δ)-channel resolvability for the worst input as*

(150) $$\begin{array}{c} S_{v}\left(\delta |W\right):=\underset{X}{\sup}S_{v}\left(\delta |X,W\right). \end{array}$$

When $W^{n}$ is the *identity mapping*, the problem of channel resolvability reduces to that of source resolvability, which has been investigated in the foregoing sections. In this sense, the problem of channel resolvability is a generalization of the problem of source resolvability.

Similarly to the problem of source resolvability, we may also use the divergence between the target output distribution $P_{Y^{n}}$ and the approximated output distribution $P_{{\tilde{Y}}^{n}}$ as an approximation measure.

**Definition** **7** (mean δ-channel resolvability: divergence)**.** *Let δ≥0 be fixed arbitrarily. A resolution rate R≥0 is said to be mean δ-achievable for X (under the divergence) if there exists a general source $\tilde{X}={\left\{{\tilde{X}}^{n}\right\}}_{n=1}^{\infty }$ satisfying*

(151) $$\begin{array}{cc} \underset{n\to \infty }{lim sup}\frac{1}{n}H\left({\tilde{X}}^{n}\right) & \leq R, \end{array}$$

(152) $$\begin{array}{cc} \underset{n\to \infty }{lim sup}D({\tilde{Y}}^{n}\left|\right|Y^{n}) & \leq \delta , \end{array}$$

*where ${\tilde{Y}}^{n}$ denotes the output via $W^{n}$ due to input ${\tilde{X}}^{n}$. The infimum of all mean δ-achievable rates for X, i.e.,*

(153) $$\begin{array}{c} {\bar{S}}_{v}^{D}\left(\delta |X,W\right):=\inf\left\{R:\;R\;is\;mean\;\delta -achievable\;for\;X\right\} \end{array}$$

*is referred to as the mean δ-channel resolvability for X. We also define the mean δ-channel resolvability for the worst input as*

(154) $$\begin{array}{c} {\bar{S}}_{v}^{D}\left(\delta |W\right):=\underset{X}{\sup}{\bar{S}}_{v}^{D}\left(\delta |X,W\right). \end{array}$$

**Definition** **8** (VL δ-channel resolvability: divergence)**.** Let δ≥0 be fixed arbitrarily. A resolution rate R≥0 is said to be VL δ-achievable or simply $v_{D}\left(\delta \right)$-achievable for X (under the divergence) if there exists a VL uniform random number $U^{\left(L_{n}\right)}$ and a deterministic mapping $\varphi_{n}:U^{*}\to X^{n}$ satisfying

(155) $$\begin{array}{cc} \underset{n\to \infty }{lim sup}\frac{1}{n}E\left[L_{n}\right] & \leq R, \end{array}$$

(156) $$\begin{array}{cc} \underset{n\to \infty }{lim sup}D({\tilde{Y}}^{n}\left|\right|Y^{n}) & \leq \delta , \end{array}$$

*where E[·] denotes the expected value and ${\tilde{Y}}^{n}$ denotes the output via $W^{n}$ due to input ${\tilde{X}}^{n}=\varphi_{n}\left(U^{\left(L_{n}\right)}\right)$. The infimum of all $v_{D}\left(\delta \right)$-achievable rates for X, i.e.,*

(157) $$\begin{array}{c} S_{v}^{D}\left(\delta |X,W\right):=\inf\left\{R:\;R\;is\;v_{D}\left(\delta \right)-achievable\;for\;X\right\} \end{array}$$

*is called the VL δ-channel resolvability or simply $v_{D}\left(\delta \right)$-channel resolvability for X. We also define the VL δ-channel resolvability or simply $v_{D}\left(\delta \right)$-channel resolvability for the worst input as*

(158) $$\begin{array}{c} S_{v}^{D}\left(\delta |W\right):=\underset{X}{\sup}S_{v}^{D}\left(\delta |X,W\right). \end{array}$$

**Remark** **12.** *Since the outputs of a deterministic mapping ${\tilde{X}}^{n}=\varphi_{n}\left(U^{\left(L_{n}\right)}\right)$ form a general source $\tilde{X}$, it holds that*

(159) $$\begin{array}{cc} {\bar{S}}_{v}\left(\delta |X,W\right) & \leq S_{v}\left(\delta |X,W\right)\;\;\;\left(\delta \in \left[0,1\right)\right), \end{array}$$

(160) $$\begin{array}{cc} {\bar{S}}_{v}^{D}\left(\delta |X,W\right) & \leq S_{v}^{D}\left(\delta |X,W\right)\;\;\left(\delta \geq 0\right) \end{array}$$

*for any general source X and general channel W. These relations lead to the analogous relation for the mean/VL δ-channel resolvability for the worst input:*

(161) $$\begin{array}{cc} {\bar{S}}_{v}\left(\delta |W\right) & \leq S_{v}\left(\delta |W\right)\;\;\;\left(\delta \in \left[0,1\right)\right), \end{array}$$

(162) $$\begin{array}{cc} {\bar{S}}_{v}^{D}\left(\delta |W\right) & \leq S_{v}^{D}\left(\delta |W\right)\;\;\left(\delta \geq 0\right). \end{array}$$

### 5.2. General Formulas

For a given general source $X={\left\{X^{n}\right\}}_{n=1}^{\infty }$ and a general channel $W={\left\{W^{n}\right\}}_{n=1}^{\infty }$, let $Y={\left\{Y^{n}\right\}}_{n=1}^{\infty }$ be the channel output via W due to input X. We define

(163) $$\begin{array}{c} H_{\left[\delta \right],W^{n}}\left(X^{n}\right)=\underset{P_{V^{n}}\in B_{\delta }\left(X^{n},W^{n}\right)}{\inf}H\left(V^{n}\right), \end{array}$$

(164) $$\begin{array}{c} H_{\left[\delta \right],W^{n}}^{D}\left(X^{n}\right)=\underset{P_{V^{n}}\in B_{\delta }^{D}\left(X^{n},W^{n}\right)}{\inf}H\left(V^{n}\right) \end{array}$$

where $H(V^{n})$ denotes the Shannon entropy of $V^{n}$ and $B_{\delta }\left(X^{n},W^{n}\right)$ and $B_{\delta }^{D}\left(X^{n},W^{n}\right)$ are defined as

(165) $$\begin{array}{cc} B_{\delta }\left(X^{n},W^{n}\right) & =\left\{P_{V^{n}}\in P\left(X^{n}\right):d\left(P_{Y^{n}},P_{Z^{n}}\right)\leq \delta \right\}, \end{array}$$

(166) $$\begin{array}{cc} B_{\delta }^{D}\left(X^{n},W^{n}\right) & =\left\{P_{V^{n}}\in P\left(X^{n}\right):D(Z^{n}\left|\right|Y^{n})\leq \delta \right\}, \end{array}$$

respectively, with $Z^{n}$ defined as the output via $W^{n}$ due to input $V^{n}$. Both $H_{\left[\delta \right],W^{n}}\left(X^{n}\right)$ and $H_{\left[\delta \right],W^{n}}^{D}\left(X^{n}\right)$ are nonincreasing functions of δ. In addition, we define

(167) $$\begin{array}{cc} H_{[\delta ],W}\left(X\right) & =\underset{n\to \infty }{lim sup}\frac{1}{n}H_{\left[\delta \right],W^{n}}\left(X^{n}\right), \end{array}$$

(168) $$\begin{array}{cc} H_{[\delta ],W}^{D}\left(X\right) & =\underset{n\to \infty }{lim sup}\frac{1}{n}H_{\left[\delta \right],W^{n}}^{D}\left(X^{n}\right), \end{array}$$

which play an important role in characterizing the mean/VL δ-channel resolvability.

We show the general formulas for the mean/VL δ-channel resolvability.

**Theorem** **5** (with variational distance)**.** *For any input process X and any general channel W,*

(169) $$\begin{array}{c} {\bar{S}}_{v}\left(\delta |X,W\right)=S_{v}\left(\delta |X,W\right)=\lim_{\gamma ↓0}H_{[\delta +\gamma ],W}\left(X\right)\;\;\;\left(\delta \in \left[0,1\right)\right). \end{array}$$

*In particular,*

(170) $$\begin{array}{cc} {\bar{S}}_{v}\left(\delta |W\right)=S_{v}\left(\delta |W\right) & =\underset{X}{\sup}\lim_{\gamma ↓0}H_{[\delta +\gamma ],W}\left(X\right)\;\;\;\left(\delta \in \left[0,1\right)\right). \end{array}$$

**Theorem** **6** (with divergence)**.** *For any input process X and any general channel W,*

(171) $$\begin{array}{c} {\bar{S}}_{v}^{D}\left(\delta |X,W\right)=S_{v}^{D}\left(\delta |X,W\right)=\lim_{\gamma ↓0}H_{[\delta +\gamma ],W}^{D}\left(X\right)\;\;\;\left(\delta \geq 0\right). \end{array}$$

*In particular,*

(172) $$\begin{array}{c} {\bar{S}}_{v}^{D}\left(\delta |W\right)=S_{v}^{D}\left(\delta |W\right)=\underset{X}{\sup}\lim_{\gamma ↓0}H_{[\delta +\gamma ],W}^{D}\left(X\right)\;\;\;\left(\delta \geq 0\right). \end{array}$$

**Remark** **13.** *It can be easily verified that the variational distance satisfies*

(173) $$\begin{array}{cc} d(P_{Y^{n}},P_{Z^{n}}) & \leq d(P_{X^{n}},P_{V^{n}}), \end{array}$$

*and, therefore, we have $B_{\delta }\left(X^{n}\right)\subseteq B_{\delta }\left(X^{n},W^{n}\right)$. This relation and formulas (32) and (169) indicate that*

(174) $$\begin{array}{c} S_{v}\left(\delta |X,W\right)\leq S_{v}\left(\delta |X\right)\;\;\;\left(\delta \in \left[0,1\right)\right) \end{array}$$

*for any given channel W. Likewise, it is well known that the divergence satisfies the data processing inequality $D({\tilde{Y}}^{n}\left|\right|Y^{n})\leq D({\tilde{X}}^{n}\left|\right|X^{n})$ [33], and formulas (101) and (171) lead to*

(175) $$\begin{array}{c} S_{v}^{D}\left(\delta |X,W\right)\leq S_{v}^{D}\left(\delta |X\right)\;\;\;\left(\delta \geq 0\right), \end{array}$$

*regardless of channel W.*

**Remark** **14.** *It is obvious that Theorems 5 and 6 reduce to Theorems 3 and 4, respectively, when the channel W is the identity mapping. Precisely, for the identity mapping W=I, the mean δ-resolvability and the v(δ)-channel resolvability for X are given by*

(176) $$\begin{array}{c} {\bar{S}}_{v}\left(\delta |X\right)=S_{v}\left(\delta |X\right)=\lim_{\gamma ↓0}H_{\left[\delta +\gamma \right]}\left(X\right), \end{array}$$

*where ${\bar{S}}_{v}\left(\delta |X\right)$ denotes the mean δ-resolvability ${\bar{S}}_{v}\left(\delta |X,W\right)$ for the identity mapping W. The analogous relationship holds under the divergence:*

(177) $$\begin{array}{c} {\bar{S}}_{v}^{D}\left(\delta |X\right)=S_{v}^{D}\left(\delta |X\right)=\lim_{\gamma ↓0}H_{\left[\delta +\gamma \right]}^{D}\left(X\right), \end{array}$$

*where ${\bar{S}}_{v}^{D}\left(\delta |X\right)$ denotes the mean δ-resolvability ${\bar{S}}_{v}^{D}\left(\delta |X,W\right)$ for the identity mapping W=I. Thus, it turns out that Theorems 5 and 6 are indeed generalizations of Theorems 3 and 4.*

**Proof** **of** **Theorems** **5** **and** **6.**

(1) Converse Part:
  Because of the general relationship (159), to prove the converse part of Theorem 5, it suffices to show that
  (178) $$\begin{array}{cc} {\bar{S}}_{v}\left(\delta |X,W\right) & \geq \lim_{\gamma ↓0}H_{[\delta +\gamma ],W}\left(X\right). \end{array}$$
  Let *R* be mean δ-achievable for X under the variational distance. Then, there exists a general source $\tilde{X}={\left\{{\tilde{X}}^{n}\right\}}_{n=1}^{\infty }$ satisfying (143) and
  (179) $$\begin{array}{c} \underset{n\to \infty }{lim sup}\delta_{n}\leq \delta , \end{array}$$
  where $\delta_{n}:=d\left(P_{Y^{n}},P_{{\tilde{Y}}^{n}}\right)$. Fixing γ>0 arbitrarily, we have $\delta_{n}\leq \delta +\gamma$ for all $n\geq n_{0}$ with some $n_{0}>0$ and then
  (180) $$\begin{array}{c} H_{\left[\delta +\gamma \right],W^{n}}\left(X^{n}\right)\leq H_{\left[\delta_{n}\right],W^{n}}\left(X^{n}\right)\;\;\;\left(n\geq n_{0}\right) \end{array}$$
  since $H_{\left[\delta \right],W^{n}}\left(X^{n}\right)$ is a nonincreasing function of δ. Since $P_{{\tilde{X}}^{n}}\in B_{\delta_{n}}\left(X^{n},W^{n}\right)$, we have $H_{\left[\delta_{n}\right],W^{n}}\left(X^{n}\right)\leq H\left({\tilde{X}}^{n}\right)$. Thus, we obtain from (143)
  (181) $$\begin{array}{c} H_{[\delta +\gamma ],W}\left(X\right)\leq \underset{n\to \infty }{lim sup}\frac{1}{n}H\left({\tilde{X}}^{n}\right)\leq R. \end{array}$$
  Since γ>0 is an arbitrary constant, this implies (178).
  The converse part of Theorem 6 can be proven in an analogous way with due modifications.
(2) Direct Part:
  Because of the general relationship (159), to prove the direct part (achievability) of Theorem 5, it suffices to show that, for any fixed γ>0, the resolution rate
  (182) $$\begin{array}{c} R=\lim_{\gamma ↓0}H_{[\delta +\gamma ],W}\left(X\right)+3\gamma \end{array}$$
  is v(δ)-achievable for X under the variational distance.
  Let $P_{V^{n}}\in B_{\delta +\gamma }\left(X^{n},W^{n}\right)$ be a source satisfying
  (183) $$\begin{array}{c} H\left(V^{n}\right)\leq H_{\left[\delta +\gamma \right],W^{n}}\left(X^{n}\right)+\gamma . \end{array}$$
  Then, by the same argument to derive (80) and (82) as developed in the proof of the direct part of Theorem 3, we can construct a VL uniform random number $U^{\left(L_{n}\right)}$ and a deterministic mapping $\varphi_{n}:U^{*}\to X^{n}$ satisfying
  (184) $$\begin{array}{c} \underset{n\to \infty }{lim sup}\frac{1}{n}E\left[L_{n}\right]\leq \lim_{\gamma ↓0}H_{[\delta +\gamma ],W}\left(X\right)+3\gamma =R \end{array}$$
  and
  (185) $$\begin{array}{c} d\left(P_{{\tilde{X}}^{n}},P_{V^{n}}\right)\leq \frac{1}{2}K^{-n\gamma }+\gamma , \end{array}$$
  where ${\tilde{X}}^{n}=\varphi_{n}\left(U^{\left(L_{n}\right)}\right)$. Let $Z^{n}$ denote the output random variable via $W^{n}$ due to input $V^{n}$. Then, letting ${\tilde{Y}}^{n}$ be the output via channel $W^{n}$ due to input ${\tilde{X}}^{n}$, we can evaluate $d(P_{{\tilde{Y}}^{n}},P_{Z^{n}})$ as
  (186) $$\begin{array}{cc} d(P_{{\tilde{Y}}^{n}},P_{Z^{n}}) & =\frac{1}{2}\sum_{y\in Y^{n}}\left|P_{{\tilde{Y}}^{n}}\left(y\right)-P_{Z^{n}}\left(y\right)\right|\\ \; & =\frac{1}{2}\sum_{y\in Y^{n}}\left|\sum_{x\in X^{n}}W\left(y|x\right)\left(P_{{\tilde{X}}^{n}}\left(x\right)-P_{V^{n}}\left(x\right)\right)\right|\\ \; & \leq \frac{1}{2}\sum_{y\in Y^{n}}\sum_{x\in X^{n}}W\left(y|x\right)\left|P_{{\tilde{X}}^{n}}\left(x\right)-P_{V^{n}}\left(x\right)\right|\\ \; & =d\left(P_{{\tilde{X}}^{n}},P_{V^{n}}\right)\leq \frac{1}{2}K^{-n\gamma }+\gamma . \end{array}$$
  Thus, we obtain
  (187) $$\begin{array}{cc} \underset{n\to \infty }{lim sup}d\left(P_{Y^{n}},P_{{\tilde{Y}}^{n}}\right) & \leq \underset{n\to \infty }{lim sup}d\left(P_{Y^{n}},P_{Z^{n}}\right)+\underset{n\to \infty }{lim sup}d\left(P_{{\tilde{Y}}^{n}},P_{Z^{n}}\right)\\ \; & \leq \delta +2\gamma , \end{array}$$
  where we have used the fact $P_{V^{n}}\in B_{\delta +\gamma }\left(X^{n},W^{n}\right)$ to derive the last inequality. Since γ>0 is an arbitrary constant, we can conclude that *R* is v(δ)-achievable for X.
  The direct part of Theorem 6 can be proven in the same way as Theorem 4 with due modifications. Fixing $P_{V^{n}}\in B_{\delta +\gamma }^{D}\left(X^{n},W^{n}\right)$ and using the encoding scheme as developed in the proof of Theorem 4, the evaluation of the average length rate is exactly the same, and we can obtain (138). A key step is to evaluate the divergence $D({\tilde{Y}}^{n}||Y^{n})$, which can be rewritten as
  (188) $$\begin{array}{c} D({\tilde{Y}}^{n}\left|\right|Y^{n})=D({\tilde{Y}}^{n}\left|\right|Z^{n})+E\left[\log\frac{P_{Z^{n}}\left({\tilde{Y}}^{n}\right)}{P_{Y^{n}}\left({\tilde{Y}}^{n}\right)}\right]. \end{array}$$
  The first term on the r.h.s. can be bounded as
  (189) $$\begin{array}{cc} D({\tilde{Y}}^{n}||Z^{n}) & \leq D({\tilde{X}}^{n}\left|\right|V^{n})\leq \frac{2\gamma }{\ln K}+\log\left(1+\frac{1}{K^{n\gamma }}\right) \end{array}$$
  as in (140), where the left inequality is due to the data processing inequality. Similarly to the derivation of (141), the second term can be bounded as
  (190) $$\begin{array}{cc} E\left[\log\frac{P_{Z^{n}}\left({\tilde{Y}}^{n}\right)}{P_{Y^{n}}\left({\tilde{Y}}^{n}\right)}\right]\; & =\sum_{y\in Y^{n}}\sum_{x\in X^{n}}P_{{\tilde{X}}^{n}}\left(x\right)W^{n}\left(y|x\right)\log\frac{P_{Z^{n}}\left(y\right)}{P_{Y^{n}}\left(y\right)}\\ \; & \leq \left(1+2\gamma \right)\left(1+\frac{1}{K^{n\gamma }}\right)\sum_{y\in Y^{n}}\sum_{x\in X^{n}}P_{V^{n}}\left(x\right)W^{n}\left(y|x\right)\log\frac{P_{Z^{n}}\left(y\right)}{P_{Y^{n}}\left(y\right)}\\ \; & =\left(1+2\gamma \right)\left(1+\frac{1}{K^{n\gamma }}\right)D(Z^{n}\left|\right|Y^{n}), \end{array}$$
  where we have used (134). Here, $D(Z^{n}||Y^{n})\leq \delta +\gamma$ because $Z^{n}$ is the output via $W^{n}$ due to input $V^{n}\in B_{\delta +\gamma }^{D}\left(X^{n},W^{n}\right)$. The rest of the steps are the same as in the proof of Theorem 4.

□

## 6. Second-Order VL Channel Resolvability

So far, we have analyzed the first-order VL resolvabilities and established various first-order resolvability theorems. One of the next important steps is the second-order analysis, and so, in this section, we generalize VL resolvabilities in the second-order setting.

### 6.1. Definitions

We now turn to considering the *second-order* resolution rates [26,27,29]. First, we consider the VL resolvability based on the variational distance.

**Definition** **9** (VL (δ,R)-channel resolvability: variational distance)**.** A second-order resolution rate L∈(−∞,+∞) is said to be VL (δ,R)-achievable (under the variational distance) for X with δ∈[0,1) if there exist a VL uniform random number $U^{\left(L_{n}\right)}$ and a deterministic mapping $\varphi_{n}:U^{*}\to X^{n}$ satisfying

(191) $$\begin{array}{cc} \underset{n\to \infty }{lim sup}\frac{1}{\sqrt{n}}\left(E[L_{n}]-nR\right) & \leq L, \end{array}$$

(192) $$\begin{array}{cc} \underset{n\to \infty }{lim sup}d\left(P_{Y^{n}},P_{{\tilde{Y}}^{n}}\right) & \leq \delta , \end{array}$$

*where ${\tilde{Y}}^{n}$ denotes the output via $W^{n}$ due to input ${\tilde{X}}^{n}=\varphi_{n}\left(U^{\left(L_{n}\right)}\right)$. The infimum of all VL (δ,R)-achievable rates for X is denoted by*

(193) $$\begin{array}{c} T_{v}\left(\delta ,R|X,W\right):=\inf\left\{L:\;L\;is\;VL\;(\delta ,R)-achievable\;for\;X\right\}. \end{array}$$

*When W is the identity mapping I, $T_{v}\left(\delta ,R|X,W\right)$ is simply denoted by $T_{v}\left(\delta ,R|X\right)$ (source resolvability).*

Next, we may consider the VL resolvability with the divergence instead of the variational distance.

**Definition** **10** (VL (δ,R)-channel resolvability: divergence). A second-order resolution rate L∈(−∞,+∞) is said to be VL (δ,R)-achievable for X (with the divergence) where δ≥0 if there exists a VL uniform random number $U^{\left(L_{n}\right)}$ and a deterministic mapping $\varphi_{n}:U^{*}\to X^{n}$ satisfying

(194) $$\begin{array}{cc} \underset{n\to \infty }{lim sup}\frac{1}{\sqrt{n}}\left(E[L_{n}]-nR\right) & \leq L, \end{array}$$

(195) $$\begin{array}{cc} \underset{n\to \infty }{lim sup}D({\tilde{Y}}^{n}\left|\right|Y^{n}) & \leq \delta , \end{array}$$

*where ${\tilde{Y}}^{n}$ denotes the output via $W^{n}$ due to input ${\tilde{X}}^{n}=\varphi_{n}\left(U^{\left(L_{n}\right)}\right)$. The infimum of all VL (δ,R)-achievable rates for X is denoted as*

(196) $$\begin{array}{c} T_{v}^{D}\left(\delta ,R|X,W\right):=\inf\left\{L:\;L\;is\;VL\;(\delta ,R)-achievable\;for\;X\right\}. \end{array}$$

*When W is the identity mapping I, $T_{v}^{D}\left(\delta ,R|X,W\right)$ is simply denoted by $T_{v}^{D}\left(\delta ,R|X\right)$ (source resolvability).*

**Remark** **15.** *It is easily verified that*

(197) $$\begin{array}{c} T_{v}\left(\delta ,R|X,W\right)=\left\{\begin{array}{cc} +\infty & for\;R<S_{v}\left(\delta |X,W\right)\\ -\infty & for\;R>S_{v}\left(\delta |X,W\right). \end{array}\right. \end{array}$$

*Hence, only the case $R=S_{v}\left(\delta |X,W\right)$ is of interest to us. The same remark also applies to $T_{v}^{D}\left(\delta ,R|X,W\right)$.*

### 6.2. General Formulas

We establish general formulas for the second-order resolvability. The proofs of the following theorems are given below subsequently to Remark 17.

**Theorem** **7** (with variational distance)**.** *For any input process X and general channel W,*

(198) $$\begin{array}{c} T_{v}\left(\delta ,R|X,W\right)=\lim_{\gamma ↓0}\underset{n\to \infty }{lim sup}\frac{1}{\sqrt{n}}\left(H_{\left[\delta +\gamma \right],W^{n}}\left(X^{n}\right)-nR\right)\;\;\;\left(\delta \in \left[0,1\right),R\geq 0\right). \end{array}$$

*In particular, in the case where W is the identity mapping I,*

(199) $$\begin{array}{c} T_{v}\left(\delta ,R|X\right)=\lim_{\gamma ↓0}\underset{n\to \infty }{lim sup}\frac{1}{\sqrt{n}}\left(H_{\left[\delta +\gamma \right]}\left(X^{n}\right)-nR\right)\;\;\;\left(\delta \in \left[0,1\right),R\geq 0\right). \end{array}$$

**Theorem** **8** (with divergence)**.** *For any input process X and general channel W,*

(200) $$\begin{array}{c} T_{v}^{D}\left(\delta ,R|X,W\right)=\lim_{\gamma ↓0}\underset{n\to \infty }{lim sup}\frac{1}{\sqrt{n}}\left(H_{\left[\delta +\gamma \right],W^{n}}^{D}\left(X^{n}\right)-nR\right)\;\;\;\left(\delta \geq 0,R\geq 0\right). \end{array}$$

*In particular, in the case where W is the identity mapping I,*

(201) $$\begin{array}{c} T_{v}^{D}\left(\delta ,R|X\right)=\lim_{\gamma ↓0}\underset{n\to \infty }{lim sup}\frac{1}{\sqrt{n}}\left(H_{\left[\delta +\gamma \right]}^{D}\left(X^{n}\right)-nR\right)\;\;\;\left(\delta \geq 0,R\geq 0\right). \end{array}$$

**Remark** **16.** *As discussed in Section 5, we may also consider using a general source $\tilde{X}$ as an input to channel W, and we can define L to be a mean (δ,R)-achievable rate for X by replacing (191) and (194) with*

(202) $$\begin{array}{cc} \underset{n\to \infty }{lim sup}\frac{1}{\sqrt{n}}\left(H({\tilde{X}}^{n})-nR\right) & \leq L. \end{array}$$

*Let ${\bar{T}}_{v}\left(\delta ,R|X,W\right)$ and ${\bar{T}}_{v}^{D}\left(\delta ,R|X,W\right)$ denote the infimum of all mean (δ,R)-achievable rates for X under the variational distance and the divergence, respectively. Then, it is not difficult to verify that*

(203) $$\begin{array}{cc} {\bar{T}}_{v}\left(\delta ,R|X,W\right) & =T_{v}\left(\delta ,R|X,W\right)\;\;\;\left(\delta \in \left[0,1\right)\right), \end{array}$$

(204) $$\begin{array}{cc} {\bar{T}}_{v}^{D}\left(\delta ,R|X,W\right) & =T_{v}^{D}\left(\delta ,R|X,W\right)\;\;\;\left(\delta \geq 0\right). \end{array}$$

*Thus, there is no loss in the (δ,R)-achievable resolution rate even if the channel input $\tilde{X}$ is restricted to be generated by the VL uniform random number $U^{\left(L_{n}\right)}$.*

**Remark** **17.** As in the first-order case, when the channel W is the identity mapping I, $T_{v}\left(\delta ,R|X\right)$ coincides with the minimum second-order length rate of the VL source codes. More precisely, we denote by $R_{v}^{*}\left(\delta ,R|X\right)$ the minimum second-order length rate of a sequence of VL source codes with the first-order average length rate R and an average error probability asymptotically not exceeding δ. Yagi and Nomura [31] have shown that

(205) $$\begin{array}{c} R_{v}^{*}\left(\delta ,R|X\right)=\lim_{\gamma ↓0}\underset{n\to \infty }{lim sup}\frac{1}{\sqrt{n}}\left(G_{\left[\delta +\gamma \right]}\left(X^{n}\right)-nR\right)\;\;\;\left(\delta \in \left[0,1\right),R\geq 0\right). \end{array}$$

*Modifying the proof of Proposition 1 (cf. Appendix C), we can show that the r.h.s. of (199) coincides with that of (205), and, therefore, it generally holds that*

(206) $$\begin{array}{c} T_{v}\left(\delta ,R|X\right)=R_{v}^{*}\left(\delta ,R|X\right)\;\;\;\left(\delta \in \left[0,1\right),R\geq 0\right). \end{array}$$

As a special case, suppose that X is a stationary and memoryless source X with the finite third absolute moment of $\log\frac{1}{P_{X}\left(X\right)}$. In this case, Kostina et al. [25] have recently given a single-letter characterization for $R_{v}^{*}\left(\delta ,R|X\right)$ with $R=H_{\left[\delta \right]}\left(X\right)=\left(1-\delta \right)H\left(X\right)$ as

(207) $$\begin{array}{c} R_{v}^{*}\left(\delta ,R|X\right)=-\sqrt{\frac{V(X)}{2\pi }}e^{-\frac{{\left(Q^{-1}\left(\delta \right)\right)}^{2}}{2}}, \end{array}$$

*where V(X) denotes the variance of $\log\frac{1}{P_{X}\left(X\right)}$ (varentropy) and $Q^{-1}$ is the inverse of the complementary cumulative distribution function of the standard Gaussian distribution. In view of the general relation (206), we can also obtain the single-letter characterization for $T_{v}\left(\delta ,R|X\right)$:*

(208) $$\begin{array}{c} T_{v}\left(\delta ,R|X\right)=-\sqrt{\frac{V(X)}{2\pi }}e^{-\frac{{\left(Q^{-1}\left(\delta \right)\right)}^{2}}{2}}. \end{array}$$

*It has not yet been made clear whether we can also have a single-letter formula for $T_{v}\left(\delta ,R|X,W\right)$ when the channel W is memoryless but not necessarily the identity mapping.*

**Proof** **of** **Theorems** **7** **and** **8.**

(1) Converse Part:
  We will show the converse part of Theorem 7. The converse part of Theorem 8 can be proved in an analogous way.
  Let *L* be VL (δ,R)-achievable for X under the variational distance. Then, there exists $U^{\left(L_{n}\right)}$ and $\varphi_{n}$ satisfying (191) and
  (209) $$\begin{array}{cc} \; & \underset{n\to \infty }{lim sup}\;\delta_{n}\leq \delta , \end{array}$$
  where we define $\delta_{n}=d\left(P_{Y^{n}},P_{{\tilde{Y}}^{n}}\right),$ and ${\tilde{Y}}^{n}$ is the output via $W^{n}$ due to input ${\tilde{X}}^{n}=\varphi_{n}\left(U^{\left(L_{n}\right)}\right)$. Equation (209) implies that, for any given γ>0, it holds that $\delta_{n}\leq \delta +\gamma$ for all $n\geq n_{0}$ with some $n_{0}>0$, and, therefore,
  (210) $$\begin{array}{c} H_{\left[\delta +\gamma \right],W^{n}}\left(X^{n}\right)\leq H_{\left[\delta_{n}\right],W^{n}}\left(X^{n}\right)\;\;\;\left(\forall n\geq n_{0}\right). \end{array}$$
  Since $P_{{\tilde{X}}^{n}}\in B_{\delta_{n}}\left(X^{n},W^{n}\right)$, we have
  (211) $$\begin{array}{c} H_{\left[\delta_{n}\right],W^{n}}\left(X^{n}\right)\leq H\left({\tilde{X}}^{n}\right). \end{array}$$
  On the other hand, it follows from (23) that
  (212) $$\begin{array}{cc} H({\tilde{X}}^{n}) & \leq H\left(U^{\left(L_{n}\right)}\right)=E\left[L_{n}\right]+H\left(L_{n}\right), \end{array}$$
  where the inequality is due to the fact that $\varphi_{n}$ is a deterministic mapping and ${\tilde{X}}^{n}=\varphi_{n}\left(U^{\left(L_{n}\right)}\right)$.
  Combining (210)–(212) yields
  (213) $$\begin{array}{cc} \; & \underset{n\to \infty }{lim sup}\frac{1}{\sqrt{n}}\left(H_{\left[\delta +\gamma \right],W^{n}}\left(X^{n}\right)-nR\right)\\ \; & \;\;\;\;\;\leq \underset{n\to \infty }{lim sup}\frac{1}{\sqrt{n}}\left(H({\tilde{X}}^{n})-nR\right)\\ \; & \;\;\;\;\;\leq \underset{n\to \infty }{lim sup}\frac{1}{\sqrt{n}}\left(E[L_{n}]-nR\right)+\underset{n\to \infty }{lim sup}\frac{1}{\sqrt{n}}H\left(L_{n}\right)\leq L, \end{array}$$
  where we have used (24) and (191) for the last inequality. Since γ>0 is arbitrary, we have
  (214) $$\begin{array}{c} \lim_{\gamma ↓0}\underset{n\to \infty }{lim sup}\frac{1}{\sqrt{n}}\left(H_{\left[\delta +\gamma \right],W^{n}}\left(X^{n}\right)-nR\right)\leq L. \end{array}$$
(2) Direct Part:
  We will show the direct part (achievability) of Theorem 7 by modifying the argument of Theorems 3 and 5, whereas the direct part of Theorem 8 can be proved in a similar manner by modifying that of Theorem 4 instead of Theorem 3.
  Letting
  (215) $$\begin{array}{c} L=\lim_{\gamma ↓0}\underset{n\to \infty }{lim sup}\frac{1}{\sqrt{n}}\left(H_{\left[\delta +\gamma \right],W^{n}}\left(X^{n}\right)-nR\right)+2\gamma , \end{array}$$
  where γ>0 is an arbitrary constant, we shall show that *L* is VL (δ,R)-achievable for X under the variational distance.
  We use the same achievability scheme as in the proof of Theorem 3 with slightly different parameter settings. For γ>0, we choose a $c_{n}>0$ so that
  (216) $$\begin{array}{c} \mathrm{Pr}\{V^{n}\notin T_{n}\}\leq \gamma \end{array}$$
  where $P_{V^{n}}\in B_{\delta +\gamma }\left(X^{n},W^{n}\right)$ with $H_{\left[\delta +\gamma \right],W^{n}}\left(X^{n}\right)+\gamma \geq H\left(V^{n}\right)$ and
  (217) $$\begin{array}{c} T_{n}:=\left\{x\in X^{n}:\frac{1}{n}\log\frac{1}{P_{V^{n}}\left(x\right)}\leq c_{n}\right\}. \end{array}$$
  We here define
  (218) $$\begin{array}{c} \ell \left(x\right):=\left\{\begin{array}{cc} \left\lceil \log\frac{1}{P_{V^{n}}\left(x\right)}+\sqrt{n}\gamma \right\rceil & for\;x\in T_{n}\\ 0 & \mathrm{otherwise} \end{array}\right. \end{array}$$
  and $\beta_{n}=\left\lceil \left(nc_{n}+\sqrt{n}\gamma \right)\right\rceil$. Arguing similarly to the proof of Theorems 3 and 5, we can show that there exist $\varphi_{n}:U^{*}\to X^{n}$ and $U^{\left(L_{n}\right)}$ such that
  (219) $$\begin{array}{c} d\left(P_{Y^{n}},P_{{\tilde{Y}}^{n}}\right)\leq \delta +2\gamma +\frac{1}{2}K^{-\sqrt{n}\gamma } \end{array}$$
  and
  (220) $$\begin{array}{cc} E[L_{n}] & \leq \left(1+\frac{1}{K^{\sqrt{n}\gamma }}\right)\left(H_{\left[\delta +\gamma \right],W^{n}}\left(X^{n}\right)+2\sqrt{n}\gamma +1\right). \end{array}$$
  Therefore, we obtain
  (221) $$\begin{array}{c} \lim_{n\to \infty }d\left(P_{Y^{n}},P_{{\tilde{Y}}^{n}}\right)\leq \delta +2\gamma \end{array}$$
  and
  (222) $$\begin{array}{cc} \; & \underset{n\to \infty }{lim sup}\frac{1}{\sqrt{n}}\left(E[L_{n}]-nR\right)\\ \; & \;\;\;\;\;\leq \underset{n\to \infty }{lim sup}\frac{1}{\sqrt{n}}\left(H_{\left[\delta +\gamma \right],W^{n}}\left(X^{n}\right)-nR\right)+2\gamma \\ \; & \;\;\;\;\;\leq \lim_{\gamma ↓0}\underset{n\to \infty }{lim sup}\frac{1}{\sqrt{n}}\left(H_{\left[\delta +\gamma \right],W^{n}}\left(X^{n}\right)-nR\right)+2\gamma =L. \end{array}$$
  Since γ>0 is arbitrary, *L* is VL (δ,R)-achievable for X.

□

## 7. Conclusions

We have investigated the problem of *VL source/channel resolvability*, in which a given target probability distribution is approximated by transforming *VL uniform random numbers*. Table 1 summarizes various first-order resolvabilities and their characterizations in terms of information quantities. In this table, the theorem numbers that contain the corresponding characterization are also indicated.

In this paper, we have first analyzed the fundamental limits on the VL δ-source resolvability with the variational distance in Theorem 3. The VL δ-source resolvability is essentially characterized in terms of smooth Shannon entropies. In the proof of the direct part, we have developed a simple method for information spectrum slicing, in which sliced information densities quantized to the same integer are approximated by an FL uniform random number of the same length. Next, we have extended the analysis to the δ-source resolvability under the unnormalized divergence in Theorem 4. The smoothed entropy with the divergence again plays an important role in characterizing the δ-source resolvability.

Then, we have addressed the problem of δ-channel resolvability. It has been revealed in Theorems 5 and 6 that using an arbitrary general source as a coin distribution (mean-resolvability problem) cannot go beyond the fundamental limits of the VL resolvability, in which only VL uniform random numbers are allowed to be a coin distribution. As in the case of source resolvability, we have discussed the δ-channel resolvability under the variational distance and the unnormalized divergence. The second-order channel resolvability has been characterized in Theorems 7 and 8 as well as the first-order case. We notice here that a counterpart of the VL uniform random number is the problem of VL source coding, for which the general treatment, focused on overflow/underflow probabilities, is found in [38]. Indeed, when the variational distance is used as an approximation measure, it turns out that the δ-*source* resolvability is equal to the minimum achievable rate of VL source codes with an error probability of less than or equal to δ. This is a parallel relationship between *FL source resolvability* and the minimum achievable rate of *FL source codes* [6,7]. It is of interest to investigate whether there is a coding problem to which the δ-*channel* resolvability is closely related.

When δ=0, asymptotically exact approximation is required. In the case where the channel W is the identity mapping I, it turned out that the source resolvability under the variational distance and the unnormalized divergence coincides and is given by $\lim_{\gamma ↓0}H_{\left[\gamma \right]}\left(X\right)$, where X is the general target source. This result is analogous to the dual problem of *VL intrinsic randomness* [5,36], in which the maximum achievable rates of VL uniform random numbers extracted from a given source X are the same under two kinds of approximation measures. It should be emphasized that in the case of VL intrinsic randomness, the use of *normalized* divergence as an approximation measure results in the same general formula as with the variational distance and the unnormalized divergence, which does not necessarily hold in the case of mean/VL resolvability (cf. Remark 11). It is also noteworthy that whereas only the case of δ=0 has been completely solved for the VL intrinsic randomness, we have also dealt with the case of a δ>0 for the VL source/channel resolvability.

When X is a stationary and memoryless source or is even with a one-point spectrum (cf. Corollary 1), the formulas established reduce to a single-letter characterization for the first- and second-order source resolvability under the variational distance. In the case where the divergence is an approximation measure and/or the channel W is a non-identity mapping, however, it has not yet been made clear whether we can derive a single-letter characterization for the δ-source/channel resolvability. This question remains to be studied.

As noted in Remark 10, the order of arguments in divergence $D({\tilde{X}}^{n}||X^{n})$ is important, and it seems difficult to extend the analyses in this paper to the case with the reversed $D(X^{n}||{\tilde{X}}^{n})$ used as an approximation measure. In the context of intrinsic randomness, the reversed divergence is also discussed [23]. Investigating the problem of source/channel resolvability using such divergence is an interesting research topic.

## Figures and Tables

**Table 1:** Summary of First-Order Resolvability and Information Quantities.

Approximation Measure	Resolvability	Characterization	Theorem #
Fixed-Length Resolvability			
Variational Distance	$S_{f}\left(X\right)$	$\bar{H}\left(X\right)$	Theorem 1 [6]
	$S_{f}\left(\delta X\right)$	${\bar{H}}_{\delta }\left(X\right)$	Theorem 2 [7]
Variable-Length Resolvability			
Variational Distance	$S_{v}\left(\delta X\right)$	$\lim_{\gamma ↓0}H_{\left[\delta +\gamma \right]}\left(X\right)$	Theorem 3
	$S_{v}\left(\delta X,W\right)$	$\lim_{\gamma ↓0}H_{[\delta +\gamma ],W}\left(X\right)$	Theorem 5
Divergence	$S_{v}^{D}\left(\delta X\right)$	$\lim_{\gamma ↓0}H_{\left[\delta +\gamma \right]}^{D}\left(X\right)$	Theorem 4
	$S_{v}^{D}\left(\delta X,W\right)$	$\lim_{\gamma ↓0}H_{[\delta +\gamma ],W}^{D}\left(X\right)$	Theorem 6

## Data Availability

Not applicable.

## Appendix A. Proof of Equation (31)

(i) We first show $\lim_{\alpha ↑1}H_{\left[\delta \right]}^{\alpha }\left(X^{n}\right)\leq H_{\left[\delta \right]}\left(X^{n}\right)$.

Fix γ>0 arbitrarily. We choose $P_{V^{n}}\in B_{\delta }\left(X^{n}\right)$ satisfying

(A1) $$\begin{array}{c} H_{\left[\delta \right]}\left(X^{n}\right)+\gamma \geq H\left(V^{n}\right). \end{array}$$

It is well known that the Rényi entropy of order α defined as

$$\begin{array}{c} H^{\alpha }\left(V^{n}\right)=\frac{1}{1-\alpha }\log\sum_{x\in X^{n}}P_{V^{n}}{\left(x\right)}^{\alpha }\;\;\;\left(\forall \alpha \in \left(0,1\right)\cup \left(1,+\infty \right)\right) \end{array}$$

satisfies

(A2) $$\begin{array}{c} H\left(V^{n}\right)=\lim_{\alpha \to 1}H^{\alpha }\left(V^{n}\right). \end{array}$$

By definition, we have

(A3) $$\begin{array}{c} H^{\alpha }\left(V^{n}\right)\geq H_{\left[\delta \right]}^{\alpha }\left(X^{n}\right)\;\;\;\left(\forall \alpha \in \left(0,1\right)\cup \left(1,+\infty \right)\right), \end{array}$$

leading to

(A4) $$\begin{array}{c} H\left(V^{n}\right)=\lim_{\alpha ↑1}H^{\alpha }\left(V^{n}\right)\geq \lim_{\alpha ↑1}H_{\left[\delta \right]}^{\alpha }\left(X^{n}\right). \end{array}$$

Combining (A1) and (A4) yields

(A5) $$\begin{array}{c} H_{\left[\delta \right]}\left(X^{n}\right)+\gamma \geq H\left(V^{n}\right)\geq \lim_{\alpha ↑1}H_{\left[\delta \right]}^{\alpha }\left(X^{n}\right). \end{array}$$

Since γ>0 is an arbitrary constant, we obtain $\lim_{\alpha ↑1}H_{\left[\delta \right]}^{\alpha }\left(X^{n}\right)\leq H_{\left[\delta \right]}\left(X^{n}\right)$.

(ii) Next, we shall show $\lim_{\alpha ↑1}H_{\left[\delta \right]}^{\alpha }\left(X^{n}\right)\geq H_{\left[\delta \right]}\left(X^{n}\right)$.

Fix γ>0 arbitrarily. We choose some $\alpha_{0}\in \left(0,1\right)$ satisfying

(A6) $$\begin{array}{c} \lim_{\alpha ↑1}H_{\left[\delta \right]}^{\alpha }\left(X^{n}\right)+\gamma \geq H_{\left[\delta \right]}^{\alpha_{0}}\left(X^{n}\right). \end{array}$$

For this $\alpha_{0}$ we choose $P_{V^{n}}\in B_{\delta }\left(X^{n}\right)$ satisfying

(A7) $$\begin{array}{c} H_{\left[\delta \right]}^{\alpha_{0}}\left(X^{n}\right)+\gamma \geq H^{\alpha_{0}}\left(V^{n}\right). \end{array}$$

Since $H^{\alpha }\left(V^{n}\right)$ is a nonincreasing function of α, we have

(A8) $$\begin{array}{c} H^{\alpha_{0}}\left(V^{n}\right)\geq H\left(V^{n}\right), \end{array}$$

and it follows from (A6)–(A8) that

(A9) $$\begin{array}{c} \lim_{\alpha ↑1}H_{\left[\delta \right]}^{\alpha }\left(X^{n}\right)+2\gamma \geq H^{\alpha_{0}}\left(V^{n}\right)\geq H\left(V^{n}\right). \end{array}$$

Since $H\left(V^{n}\right)\geq H_{\left[\delta \right]}\left(X^{n}\right)$, due to $P_{V^{n}}\in B_{\delta }\left(X^{n}\right)$, and γ>0 is arbitrarily fixed, we obtain the desired inequality.

## Appendix B. Proof of Equation (34)

To prove the alternative formula (34) for the v(δ)-resolvability $S_{v}\left(\delta |X\right)$, we shall show

(A10) $$\begin{array}{c} \lim_{\gamma ↓0}H_{\left[\delta +\gamma \right]}\left(X\right)=\underset{V\in B_{\delta }\left(X\right)}{\inf}H\left(V\right)\;\;\;\left(\delta \in \left[0,1\right)\right). \end{array}$$

(i) We first show $\lim_{\gamma ↓0}H_{\left[\delta +\gamma \right]}\left(X\right)\leq \underset{V\in B_{\delta }\left(X\right)}{\inf}H\left(V\right)$.

Fix γ>0 arbitrarily. We choose $\tilde{V}={\left\{{\tilde{V}}^{n}\right\}}_{n=1}^{\infty }\in B_{\delta }\left(X\right)$ satisfying

(A11) $$\begin{array}{c} H\left(\tilde{V}\right)\leq \underset{V\in B_{\delta }\left(X\right)}{\inf}H\left(V\right)+\gamma . \end{array}$$

For $\tilde{V}\in B_{\delta }\left(X\right)$, we have $d(X^{n},{\tilde{V}}^{n})\leq \delta +\gamma$ for all $n\geq n_{0}$ with some $n_{0}>0$, yielding

(A12) $$\begin{array}{c} H_{\left[\delta +\gamma \right]}\left(X^{n}\right)\leq H\left({\tilde{V}}^{n}\right)\;\;\;\left(\forall n\geq n_{0}\right). \end{array}$$

Thus, it follows from (A11) and (A12) that

(A13) $$\begin{array}{c} H_{\left[\delta +\gamma \right]}\left(X\right)\leq \underset{V\in B_{\delta }\left(X\right)}{\inf}H\left(V\right)+\gamma . \end{array}$$

Since γ>0 is an arbitrary constant, letting γ↓0 on both sides yields the desired inequality.

(ii) Next, we shall show $\lim_{\gamma ↓0}H_{\left[\delta +\gamma \right]}\left(X\right)\geq \underset{V\in B_{\delta }\left(X\right)}{\inf}H\left(V\right)$.

Fix λ>0 arbitrarily. We choose an arbitrary decreasing sequence of positive numbers ${\left\{\gamma_{i}\right\}}_{i=1}^{\infty }$ satisfying $\gamma_{1}>\gamma_{2}>\cdots \to 0$. Then, we have

(A14) $$\begin{array}{c} \lim_{\gamma ↓0}H_{\left[\delta +\gamma \right]}\left(X\right)=\lim_{i\to \infty }H_{\left[\delta +\gamma_{i}\right]}\left(X\right). \end{array}$$

Additionally, by the definition of the limit superior, for each i=1,2,⋯ we have

(A15) $$\begin{array}{c} \frac{1}{n}H_{\left[\delta +\gamma_{i}\right]}\left(X^{n}\right)\leq H_{\left[\delta +\gamma_{i}\right]}\left(X\right)+\lambda \;\;\;\left(\forall n\geq n_{i}\right) \end{array}$$

with some $0<n_{1}<n_{2}<\cdots$. Now, for each n=1,2,⋯, we denote by $i_{n}$ the index *i* satisfying

(A16) $$\begin{array}{c} n_{i}\leq n<n_{i+1}. \end{array}$$

Then, from (A15), we obtain

(A17) $$\begin{array}{c} \frac{1}{n}H_{\left[\delta +\gamma_{i_{n}}\right]}\left(X^{n}\right)\leq H_{\left[\delta +\gamma_{i_{n}}\right]}\left(X\right)+\lambda \;\;\;\left(\forall n\geq n_{1}\right). \end{array}$$

On the other hand, by the definition of $H_{\left[\delta +\gamma_{i_{n}}\right]}\left(X^{n}\right)$, for each n=1,2,⋯, we can choose some $V_{i_{n}}^{n}\in B_{\delta +\gamma_{i_{n}}}\left(X^{n}\right)$ satisfying

(A18) $$\begin{array}{c} \frac{1}{n}H\left(V_{i_{n}}^{n}\right)\leq \frac{1}{n}H_{\left[\delta +\gamma_{i_{n}}\right]}\left(X^{n}\right)+\lambda . \end{array}$$

We now construct the general source $\tilde{V}={\left\{V_{i_{n}}^{n}\right\}}_{n=1}^{\infty }$. Since $V_{i_{n}}^{n}\in B_{\delta +\gamma_{i_{n}}}\left(X^{n}\right)$ for all $n\geq n_{1}$ indicates that

(A19) $$\begin{array}{c} \underset{n\to \infty }{lim sup}d\left(X^{n},V_{i_{n}}^{n}\right)\leq \delta +\lim_{n\to \infty }\gamma_{i_{n}}=\delta , \end{array}$$

the general source satisfies $\tilde{V}\in B_{\delta }\left(X\right)$.

From (A17) and (A18), we obtain

(A20) $$\begin{array}{c} \frac{1}{n}H\left(V_{i_{n}}^{n}\right)\leq H_{\left[\delta +\gamma_{i_{n}}\right]}\left(X\right)+2\lambda \;\;\;\left(\forall n\geq n_{1}\right). \end{array}$$

In view of (A14) and the fact $\tilde{V}\in B_{\delta }\left(X\right)$, taking $\underset{n\to \infty }{lim sup}$ on both sides yields

(A21) $$\begin{array}{cc} \underset{V\in B_{\delta }\left(X\right)}{\inf}H\left(V\right)\leq H\left(\tilde{V}\right) & \leq \underset{n\to \infty }{lim sup}H_{\left[\delta +\gamma_{i_{n}}\right]}\left(X\right)+2\lambda \\ \; & =\lim_{\gamma ↓0}H_{\left[\delta +\gamma \right]}\left(X\right)+2\lambda . \end{array}$$

Since λ>0 is an arbitrary constant, letting λ↓0 yields the desired inequality.

## Appendix C. Proof of Proposition 1

We shall prove the equality and inequality in (40). Equation (41) is an immediate consequence of (40) because ${\bar{H}}_{\delta }\left(X\right)$ is a right-continuous function [2].

(i) We first shall show the equality in (40): $H_{\left[\delta \right]}\left(X\right)=G_{\left[\delta \right]}\left(X\right)$.

We show $H_{\left[\delta \right]}\left(X\right)\leq G_{\left[\delta \right]}\left(X\right)$. For any given γ>0 and $P_{X^{n}}$, let $A_{n}^{*}\subseteq X^{n}$ be a subset of $X^{n}$ which satisfies

(A22) $$\begin{array}{c} \mathrm{Pr}\{X^{n}\in A_{n}^{*}\}\geq 1-\delta \end{array}$$

and

(A23) $$\begin{array}{c} G_{\left[\delta \right]}\left(X^{n}\right)+\gamma \geq \sum_{x\in A_{n}^{*}}P_{X^{n}}\left(x\right)\log\frac{1}{P_{X^{n}}\left(x\right)}=:F\left(A_{n}^{*}\right). \end{array}$$

Choose $x_{0}\in X^{n}\setminus A_{n}^{*}$ arbitrarily. Set $P_{{\tilde{X}}^{n}}$ so that

(A24) $$\begin{array}{c} P_{{\tilde{X}}^{n}}\left(x\right)=\left\{\begin{array}{cc} P_{X^{n}}\left(x\right) & for\;x\in A_{n}^{*}\\ \alpha_{0} & for\;x=x_{0}\\ 0 & \mathrm{otherwise}, \end{array}\right. \end{array}$$

where we define $\alpha_{0}=\mathrm{Pr}\left\{X^{n}\notin A_{n}^{*}\right\}$.

The variational distance between $P_{X^{n}}$ and $P_{{\tilde{X}}^{n}}$ satisfies

(A25) $$\begin{array}{cc} d(P_{X^{n}},P_{{\tilde{X}}^{n}}) & =\frac{1}{2}\sum_{x\notin A_{n}^{*}}\left|P_{X^{n}}\left(x\right)-P_{{\tilde{X}}^{n}}\left(x\right)\right|\\ \; & \leq \frac{1}{2}\sum_{x\notin A_{n}^{*}}\left(P_{X^{n}}\left(x\right)+P_{{\tilde{X}}^{n}}\left(x\right)\right)\leq \alpha_{0}=1-\mathrm{Pr}\left\{X^{n}\in A_{n}^{*}\right\}\leq \delta , \end{array}$$

where the last inequality is due to (A22). Therefore, $P_{{\tilde{X}}^{n}}\in B_{\delta }\left(X^{n}\right)$, and this implies

(A26) $$\begin{array}{cc} H_{\left[\delta \right]}\left(X^{n}\right)\leq H\left({\tilde{X}}^{n}\right) & =F\left(A_{n}^{*}\right)+\alpha_{0}\log\frac{1}{\alpha_{0}}\\ \; & \leq G_{\left[\delta \right]}\left(X^{n}\right)+\alpha_{0}\log\frac{1}{\alpha_{0}}+\gamma \\ \; & \leq G_{\left[\delta \right]}\left(X^{n}\right)+\frac{\log e}{e}+\gamma , \end{array}$$

where the first inequality is due to (A23) and the last inequality is due to the inequality $x\log x\geq -\frac{\log e}{e}$ for all x>0. Thus, we obtain the desired inequality: $H_{\left[\delta \right]}\left(X\right)\leq G_{\left[\delta \right]}\left(X\right)$.

Next, we shall show $H_{\left[\delta \right]}\left(X\right)\geq G_{\left[\delta \right]}\left(X\right)$. Assume, without loss of generality, that the elements of $X^{n}$ are indexed as $x_{1},x_{2},\cdots \in X^{n}$ so that

(A27) $$\begin{array}{c} P_{X^{n}}\left(x_{i}\right)\geq P_{X^{n}}\left(x_{i+1}\right)\;\;\;\left(\forall i=1,2,\cdots \right). \end{array}$$

For a given δ∈[0,1), let $j^{*}$ denote the integer satisfying

(A28) $$\begin{array}{c} \sum_{i=1}^{j^{*}-1}P_{X^{n}}\left(x_{i}\right)<1-\delta ,\;\;\;\;\;\sum_{i=1}^{j^{*}}P_{X^{n}}\left(x_{i}\right)\geq 1-\delta . \end{array}$$

Let $V_{\delta }^{n}$ be a random variable taking values in $X^{n}$ whose probability distribution is given by

(A29) $$\begin{array}{c} P_{V_{\delta }^{n}}\left(x_{i}\right)=\left\{\begin{array}{cc} P_{X^{n}}\left(x_{i}\right)+\delta & \mathrm{for}\;i=1\\ P_{X^{n}}\left(x_{i}\right) & \mathrm{for}\;i=2,3,\cdots ,j^{*}-1\\ P_{X^{n}}\left(x_{i}\right)-\varepsilon_{n} & \mathrm{for}\;i=j^{*}\\ 0 & \mathrm{otherwise}, \end{array}\right. \end{array}$$

where $\varepsilon_{n}:=\delta -\sum_{i\geq j^{*}+1}P_{X^{n}}\left(x_{i}\right)$. It is easily checked that $0\leq \varepsilon_{n}\leq P_{X^{n}}\left(x_{j^{*}}\right)$ and the probability distribution $P_{V_{\delta }^{n}}$ *majorizes* (for a sequence $u=(u_{1},u_{2},\cdots ,u_{L})$ of length *L*, we denote by $\tilde{u}=\left({\tilde{u}}_{1},{\tilde{u}}_{2},\cdots ,{\tilde{u}}_{L}\right)$ a permuted version of u satisfying ${\tilde{u}}_{i}\geq {\tilde{u}}_{i+1}$ for all i=1,2,⋯,L−1, where ties are arbitrarily broken. We say $u=(u_{1},u_{2},\cdots ,u_{L})$ *majorizes* $v=(v_{1},v_{2},\cdots ,v_{L})$ if $\sum_{i=1}^{j}{\tilde{u}}_{i}\geq \sum_{i=1}^{j}{\tilde{v}}_{i}$ for all j=1,2,⋯,L.) any $P_{V^{n}}\in B_{\delta }\left(X^{n}\right)$ [39]. Since the Shannon entropy is a *Schur concave* function (The function f(u) is said to be *Schur concave* if f(u)≤f(v) for any pair (u,v), where v is majorized by u.) According to [40], we immediately obtain the following lemma, which is of use to compute $H_{\left[\delta \right]}\left(X^{n}\right)$.

**Lemma** **A1** (Ho and Yeung [39]).

(A30) $$\begin{array}{c} H_{\left[\delta \right]}\left(X^{n}\right)=H\left(V_{\delta }^{n}\right)\;\;\;\left(\forall \delta \in \left[0,1\right)\right). \end{array}$$

By the definition of $G_{\left[\delta \right]}\left(X^{n}\right)$, we obtain

(A31) $$\begin{array}{cc} G_{\left[\delta \right]}\left(X^{n}\right) & \leq \sum_{i=1}^{j^{*}}P_{X^{n}}\left(x_{i}\right)\log\frac{1}{P_{X^{n}}\left(x_{i}\right)} \end{array}$$

(A32) $$\begin{array}{cc} \; & \leq H\left(V_{\delta }^{n}\right)+P_{X^{n}}\left(x_{1}\right)\log\frac{1}{P_{X^{n}}\left(x_{1}\right)}+P_{X^{n}}\left(x_{j^{*}}\right)\log\frac{1}{P_{X^{n}}\left(x_{j^{*}}\right)} \end{array}$$

(A33) $$\begin{array}{cc} \; & \leq H\left(V_{\delta }^{n}\right)+\frac{2\log e}{e}, \end{array}$$

where the last inequality is due to $x\log x\geq -\frac{\log e}{e}$ for all x>0. Thus, it follows from Lemma A1 that

(A34) $$\begin{array}{cc} G_{\left[\delta \right]}\left(X\right) & \leq \underset{n\to \infty }{lim sup}\frac{1}{n}H\left(V_{\delta }^{n}\right)=H_{\left[\delta \right]}\left(X\right), \end{array}$$

which is the desired inequality.

(ii) Next, we show the inequality in (40): $H_{\left[\delta \right]}\left(X\right)\leq \left(1-\delta \right){\bar{H}}_{\delta -\gamma }\left(X\right)$. By the definition of ${\bar{H}}_{\delta -\gamma }\left(X\right)$, for any η>0, there exists some $n_{0}>0$ such that

(A35) $$\begin{array}{c} \mathrm{Pr}\left\{X^{n}\in T_{n}\right\}\geq 1-\delta \;\;\;\left(\forall n\geq n_{0}\right), \end{array}$$

where we define

(A36) $$\begin{array}{c} T_{n}=\left\{x\in X^{n}:\frac{1}{n}\log\frac{1}{P_{X^{n}}\left(x\right)}\leq {\bar{H}}_{\delta -\gamma }\left(X\right)+\eta \right\}. \end{array}$$

Choose a sequence $x_{0}\in T_{n}$ arbitrarily. Set $P_{V^{n}}$ so that

(A37) $$\begin{array}{c} P_{V^{n}}\left(x\right)=\left\{\begin{array}{cc} \alpha_{n}P_{X^{n}}\left(x\right)+\delta & for\;x=x_{0}\\ \alpha_{n}P_{X^{n}}\left(x\right) & for\;x\neq x_{0},x\in T_{n}\\ 0 & \mathrm{otherwise}, \end{array}\right. \end{array}$$

where we define $\alpha_{n}=\left(1-\delta \right)/\mathrm{Pr}\left\{X^{n}\in T_{n}\right\}$. Then, the variational distance between $P_{X^{n}}$ and $P_{V^{n}}$ satisfies

(A38) $$\begin{array}{cc} d(P_{X^{n}},P_{V^{n}}) & \leq \frac{\delta }{2}+\frac{1}{2}\sum_{x\in T_{n}}P_{X^{n}}\left(x\right)\left|1-\alpha_{n}\right|+\frac{1}{2}\sum_{x\in T_{n}^{c}}P_{X^{n}}\left(x\right)\\ \; & =\frac{\delta }{2}+\frac{1}{2}\mathrm{Pr}\left\{X^{n}\in T_{n}\right\}\left(1-\alpha_{n}\right)+\mathrm{Pr}\left\{X^{n}\in T_{n}^{c}\right\}\\ \; & =\delta , \end{array}$$

which indicates that $P_{V^{n}}\in B_{\delta }\left(X^{n}\right)$. The normalized Shannon entropy $\frac{1}{n}H\left(V^{n}\right)$ can be upper bounded as

(A39) $$\begin{array}{cc} \frac{1}{n}H\left(V^{n}\right) & =\frac{1}{n}\left(\alpha_{n}P_{X^{n}}\left(x_{0}\right)+\delta \right)\log\frac{1}{\alpha_{n}P_{X^{n}}\left(x_{0}\right)+\delta }\\ \; & \;\;\;\;\;+\frac{1}{n}\sum_{x\in T_{n}\setminus \left\{x_{0}\right\}}P_{V^{n}}\left(x\right)\log\frac{1}{P_{V^{n}}\left(x\right)}\\ \; & \leq \frac{\delta }{n}\log\frac{1}{\delta }+\frac{1}{n}\sum_{x\in T_{n}}\alpha_{n}P_{X^{n}}\left(x\right)\log\frac{1}{\alpha_{n}P_{X^{n}}\left(x\right)}\\ \; & =\frac{\delta }{n}\log\frac{1}{\delta }+\frac{\alpha_{n}}{n}\sum_{x\in T_{n}}P_{X^{n}}\left(x\right)\left(\log\frac{1}{\alpha_{n}}+\log\frac{1}{P_{X^{n}}\left(x\right)}\right)\\ \; & \leq \frac{\delta }{n}\log\frac{1}{\delta }+\frac{\left(1-\delta \right)}{n}\log\frac{1}{1-\delta }+\alpha_{n}\sum_{x\in T_{n}}P_{X^{n}}\left(x\right)\left({\bar{H}}_{\delta -\gamma }\left(X\right)+\eta \right)\\ \; & =\frac{1}{n}h_{2}\left(\delta \right)+\left(1-\delta \right)\left({\bar{H}}_{\delta -\gamma }\left(X\right)+\eta \right), \end{array}$$

where we have used the fact that $\frac{1}{n}\log\frac{1}{P_{X^{n}}\left(x\right)}\leq {\bar{H}}_{\delta -\gamma }\left(X\right)+\eta$ for $x\in T_{n}$ to obtain the second inequality, and $h_{2}\left(\delta \right):=-\delta \log\delta -\left(1-\delta \right)\log\left(1-\delta \right)$ denotes the binary entropy function. On the other hand, because $P_{V^{n}}\in B_{\delta }\left(X^{n}\right)$, there exists some $n_{1}>0$ such that

(A40) $$\begin{array}{c} H_{\left[\delta \right]}\left(X\right)\leq \frac{1}{n}H\left(V^{n}\right)+\eta \;\;\;\left(\forall n>n_{1}\right). \end{array}$$

Combining (A39) and (A40) yields

(A41) $$\begin{array}{c} H_{\left[\delta \right]}\left(X\right)\leq \left(1-\delta \right){\bar{H}}_{\delta -\gamma }\left(X\right)+3\eta , \end{array}$$

and since η>0 is arbitrarily fixed, (A41) means the desired inequality.

## Appendix D. Proof of Corollary 1

We first show (44), i.e.,

(A42) $$\begin{array}{c} S_{f}\left(\delta |X\right)\overset{\left(a\right)}{=}{\bar{H}}_{\delta }\left(X\right)\overset{\left(b\right)}{=}H^{*}\left(X\right). \end{array}$$

Equality (a) is due to Theorem 2. To prove equality (b), we notice that

(A43) $$\begin{array}{c} \underline{H}\left(X\right)\leq {\bar{H}}_{\delta }\left(X\right)\leq \bar{H}\left(X\right)\;\;\;\left(\forall \delta \in \left[0,1\right)\right) \end{array}$$

for general source X by definition. If source X is with one-point spectrum, the left-hand side (l.h.s.) and the r.h.s. in (A43) match, i.e., $H^{*}\left(X\right):=\underline{H}\left(X\right)=\bar{H}\left(X\right)$, and the squeeze theorem indicates equality (b).

Next, we show (45), i.e.,

(A44) $$\begin{array}{c} S_{v}\left(\delta |X\right)\overset{\left(c\right)}{=}H_{\left[\delta \right]}\left(X\right)\overset{\left(d\right)}{=}G_{\left[\delta \right]}\left(X\right)\overset{\left(e\right)}{=}\left(1-\delta \right)H^{*}\left(X\right). \end{array}$$

Equality (d) is a direct consequence of Proposition 1. To prove equality (e), we notice a general relationship

(A45) $$\begin{array}{c} \left(1-\delta \right)\underline{H}\left(X\right)\leq G_{\left[\delta \right]}\left(X\right)\leq \left(1-\delta \right)\bar{H}\left(X\right)\;\;\;\left(\forall \delta \in \left[0,1\right)\right) \end{array}$$

for general source X, where the first inequality is due to ([35], Theorem 4) and the second one is due to Proposition 1 (cf. Equation (42)). In view of $H^{*}\left(X\right)=\underline{H}\left(X\right)=\bar{H}\left(X\right)$ for source X with one-point spectrum, the squeeze theorem for (A45) indicates equality (e), i.e.,

(A46) $$\begin{array}{c} G_{\left[\delta \right]}\left(X\right)=\left(1-\delta \right)H^{*}\left(X\right)\;\;\;\left(\forall \delta \in \left[0,1\right)\right). \end{array}$$

The r.h.s. of (A46) is obviously right-continuous in δ∈[0,1), and so is the l.h.s. side $G_{\left[\delta \right]}\left(X\right)=H_{\left[\delta \right]}\left(X\right)$ if source X is with one-point spectrum. The right-continuity of $H_{\left[\delta \right]}\left(X\right)$ and Theorem 3 indicate equality (c).

## Appendix E. Proof of Lemma 1

We first show (99). For two general sources $X={\left\{X^{n}\right\}}_{n=1}^{\infty }$ and $\tilde{X}={\left\{{\tilde{X}}^{n}\right\}}_{n=1}^{\infty }$, the following well-known inequality (cf. ([33], Problem 3.18)) holds between the variational distance and the divergence:

(A47) $$\begin{array}{c} \frac{2{\left(d(P_{X^{n}},P_{{\tilde{X}}^{n}})\right)}^{2}}{\ln K}\leq D({\tilde{X}}^{n}\left|\right|X^{n}). \end{array}$$

This inequality implies that any $P_{V^{n}}\in B_{g(\delta )}^{D}\left(X^{n}\right)$ satisfies $P_{V^{n}}\in B_{\delta }\left(X^{n}\right)$. Thus, we have

(A48) $$\begin{array}{c} H_{\left[\delta \right]}\left(X^{n}\right)\leq H_{\left[g(\delta )\right]}^{D}\left(X^{n}\right). \end{array}$$

Now, we shall show the rightmost equality of (100). It obviously follows from (99) that

(A49) $$\begin{array}{c} \lim_{\delta ↓0}H_{\left[\delta \right]}\left(X\right)\leq \lim_{\delta ↓0}H_{\left[\delta \right]}^{D}\left(X\right). \end{array}$$

To show the opposite inequality, in view of (41), it suffices to show

(A50) $$\begin{array}{c} \lim_{\delta ↓0}G_{\left[\delta \right]}\left(X\right)\geq \lim_{\delta ↓0}H_{\left[\delta \right]}^{D}\left(X\right). \end{array}$$

Fix δ∈(0,1) and γ>0 arbitrarily. We choose $A_{n}\subseteq X^{n}$ satisfying

(A51) $$\begin{array}{cc} G_{\left[\delta \right]}\left(X^{n}\right)+\gamma & \geq \sum_{x\in A_{n}}P_{X^{n}}\left(x\right)\log\frac{1}{P_{X^{n}}\left(x\right)}, \end{array}$$

(A52) $$\begin{array}{cc} \alpha_{0} & :=\mathrm{Pr}\{X^{n}\in A_{n}\}\geq 1-\delta . \end{array}$$

We arrange a new random variable $V^{n}$ subject to

(A53) $$\begin{array}{c} P_{V^{n}}\left(x\right)=\left\{\begin{array}{cc} \frac{P_{X^{n}}\left(x\right)}{\alpha_{0}} & if\;x\in A_{n}\\ 0 & \mathrm{otherwise}. \end{array}\right. \end{array}$$

Then, we obtain

(A54) $$\begin{array}{c} D(V^{n}\left|\right|X^{n})=\sum_{x\in A_{n}}P_{V^{n}}\left(x\right)\log\frac{P_{V^{n}}\left(x\right)}{P_{X^{n}}\left(x\right)}=\log\frac{1}{\alpha_{0}}\leq \log\frac{1}{1-\delta }, \end{array}$$

and, thus, letting $h\left(\delta \right)=\log\frac{1}{1-\delta }$, it holds that $P_{V^{n}}\in B_{h(\delta )}^{D}\left(X^{n}\right)$. We can expand (A51) as

(A55) $$\begin{array}{cc} G_{\left[\delta \right]}\left(X^{n}\right)+\gamma & \geq \alpha_{0}\sum_{x\in A_{n}}P_{V^{n}}\left(x\right)\log\frac{1}{\alpha_{0}P_{V^{n}}\left(x\right)}\\ \; & \geq \alpha_{0}H\left(V^{n}\right)\\ \; & \geq \left(1-\delta \right)H_{\left[h(\delta )\right]}^{D}\left(X^{n}\right), \end{array}$$

where the last inequality is due to (A52) and $P_{V^{n}}\in B_{h(\delta )}^{D}\left(X^{n}\right)$. Thus, as γ>0 is arbitrary,

(A56) $$\begin{array}{c} G_{\left[\delta \right]}\left(X\right)\geq \left(1-\delta \right)H_{\left[h(\delta )\right]}^{D}\left(X\right). \end{array}$$

Since δ∈(0,1) is arbitrary, in view of $\lim_{\delta ↓0}h\left(\delta \right)=0$, we obtain (A50).
